# Old world camels in Germany: parasitic nematode communities characterized by nemabiome analysis showed reduced anthelmintic efficacy according to the fecal egg count reduction test

**DOI:** 10.1186/s13071-025-06930-9

**Published:** 2025-07-24

**Authors:** Jenny Brachmann, Stefan Fiedler, Hannah Fischer, Jennifer S. Schmidt, Renate Radek, Georg von Samson-Himmelstjerna, Jürgen Krücken

**Affiliations:** 1 Institute for Parasitology and Tropical Veterinary Medicine, Freie Universität Berlin, Berlin, Germany; 2 Veterinary Centre for Resistance Research, Freie Universität Berlin, Berlin, Germany; 3 Federal Office of Consumer Protection and Food Safety, Berlin, Germany; 4 Evolutionary Biology, Institute of Biology, Freie Universität Berlin, Berlin, Germany; 5 Present Address: German Federal Institute of Risk Assessment, Max-Dohrn-Str. 8-10, 10589 Berlin, Germany

**Keywords:** Old world camels, Nemabiome, Gastrointestinal nematodes, Anthelmintic resistance, *Haemonchus contortus*, *Trichostrongylus colubriformis*

## Abstract

**Background:**

Gastrointestinal nematodes pose a significant health risk to grazing livestock and cause economic losses, which are further increased by anthelmintic resistance. This study examined the gastrointestinal parasite fauna of Old World Camels (OWCs) in Germany and evaluated the efficacy of anthelmintic treatment.

**Methods:**

In total, nine German OWC-keeping farms that dewormed their stock in spring 2023 were examined. The farms with their veterinarians independently selected the drug for treatment. The number of strongyle eggs per gram (EPG) feces was determined in 107 OWCs, *Camelus bactrianus* (86.0%), *Camelus dromedarius* (6.5%), and hybrids (7.5%), using the FLOTAC method (multiplication factor = 1) before and 14 days after treatment (paired sample size: 100 OWCs). The fecal egg count reduction (FECR) was calculated using bayescount and eggCounts software. For the identification and relative quantification of strongyle species, deep amplicon sequencing (nemabiome analysis) was used.

**Results:**

Farms differed widely regarding egg shedding intensities and prevalence. On most farms, the weight of the animals was only estimated. Evaluation of the anthelmintic efficacy revealed FECRs of 26.6–90.8% after treatment with albendazole, fenbendazole, ivermectin, moxidectin, or doramectin, while only on one farm treatment with monepantel resulted in > 99% FECR. The strongyle species diversity, as determined using the nemabiome approach, was low. With *Trichostrongylus colubriformis*, *Haemonchus contortus*, *Trichostrongylus axei*, and *Cooperia oncophora* abundant strongyles of German domestic ruminants dominated, while *Camelostrongylus mentulatus* also occurred. After deworming, strongyle communities almost completely consisted of *T. colubriformis* and *H. contortus*. In contrast, *C. mentulatus* and *C. oncophora* were consistently eliminated by treatments.

**Conclusions:**

This study shows the insufficient efficacy of standard treatments chosen by farmers/veterinarians for OWCs in Germany. Since treatment eliminated some species but did not eliminate others, not underdosing but resistant nematode species presumably led to treatment failure. However, owing to the small sample size, assessment of animal weight only by visual estimation, and no drugs licensed for OWCs in Europe, the term resistance should be used with care. The species *T. colubriformis* and *H. contortus* that survived after anthelmintic treatment are also frequently resistant in ruminants in Germany.

**Graphical Abstract:**

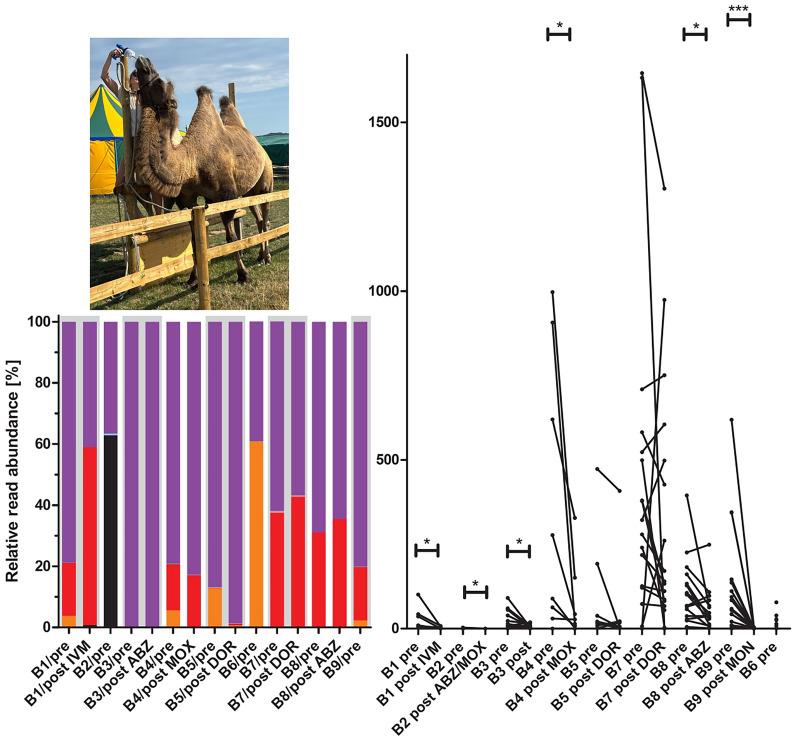

**Supplementary Information:**

The online version contains supplementary material available at 10.1186/s13071-025-06930-9.

## Background

The family of camelids (Camelidae) belongs to the order of Cetartiodactyla and is the only extant representative of the suborder Tylopoda [1, 2]. The Camelidae are divided into the group of Old World camels (OWCs) (also: large camels, Camelini) and the group of humpless New World camels (also: small camels, Lamini). Camelidae have a three-chambered stomach, which evolved convergently to the four-chambered stomach of ruminants. The OWCs include two domesticated species, *Camelus dromedarius* (Linnaeus, 1758) (dromedary; one-humped), *Camelus bactrianus* (Linnaeus, 1758) (Bactrian camel; two-humped), and the wild Bactrian camel *Camelus ferus* (Przewalski, 1878) [1, 3]. The close phylogenetic relationship allows OWC species to cross with each other, and such hybrids have been produced on purpose since they are larger than either of the parental species and can carry heavier loads [1].

Humans, especially those in steppe and desert regions, have used OWCs as versatile livestock for survival in areas with harsh environmental conditions for thousands of years. These domestic animals are valuable sources of meat, milk, skin, and wool. Old World camels are also suitable as load carriers for long trade routes. These routes brought dromedaries from North to East Africa and the Arabian Peninsula and Bactrians from Central Asia to Europe [4, 5]. Old World Camel populations are currently growing worldwide [6], and the number of individuals for both domestic species together is estimated at more than 40 million animals [7]. In Europe, OWCs are nowadays kept primarily in circuses, zoos, for tourism, but also as private leisure animals. Therefore, herds are often small and rarely exceed 20 individuals. In Germany, there is just one large farm that owns more than 100 OWCs, which are used for breeding and, since 2020, for producing milk.

Husbandry of OWCs in non-arid areas entails not only a change in environmental conditions, such as temperature, humidity, and food source, but also a change in potential health risks. This also concerns infections with parasites that have free-living stages, which strongly depend on environmental conditions influencing the survival of free-living stages [4, 8, 9]. Nematode species parasitizing OWCs vary according to region and climate and can also be dependent on the local climate conditions. Old World camels are most likely exposed to different nematode species when kept in Europe compared with the parasite fauna in arid climates in their original habitats. In addition to the new parasite species, husbandry conditions, such as herd composition and space available, also influence the risk of infection [10]. Old World camels are often kept together on the same pastures with ruminants, which results in the risk of infection of OWCs with typical ruminant parasites. In the countries of origin, OWCs graze on large areas to obtain their daily food intake. Keeping them at high stocking densities leads to increased infection pressure [11].

Gastrointestinal parasitic nematodes can affect the health and productivity (e.g., growth rate, fertility, milk production) of OWCs [12]. Most nematodes found in Camelidae are euryxenous parasites, such as *Haemonchus contortus* and *Camelostrongylus mentulatus*, which infect hosts from many different ruminant families (e.g., Bovidae, Cervidae) but can also parasitize the camelids in the suborder Tylopoda [13]. In addition, there are some stenoxenous nematodes, such as *Physocephalus dromedarii*, *Nematodirus dromedarii*, *Nematodirus mauritanicus*, *Nematodirella dromedarii*, and *Nematodirella cameli*, which only infect OWCs [4, 11, 13, 14]. Other species, such as *Haemonchus longistipes*, show a strong preference for OWCs but can also be found in ruminants [15].

Gastrointestinal nematode (GIN) infections of livestock are often caused by communities of several nematode species [16]. Characterization of these nematode communities on the individual animal or farm level has been tremendously facilitated by applying deep amplicon sequencing of PCRs targeting the internal transcribed spacer 2 (ITS-2) to obtain nemabiome data [16–18]. The method is based on a pan-strongyle PCR that has been used for decades to identify nematodes using Sanger sequencing [19]. PCR products are sequenced applying an Illumina MiSeq [17] or a PacBio [20] next generation sequencing approach. Using a barcoding technique, PCR products originating from different samples can be pooled, and data of individual samples can then be demultiplexed and assigned to individual samples again by bioinformatic means [18]. Owing to copy number variation of the ITS-2 region in the genome between species and the different number of cells in third stage larvae, for which the method was initially developed, correction factors were established for the most important strongyle parasites of cattle and small ruminants [17, 21]. However, these correction factors are only available for the most abundant and clinically important strongyle species. For other species, this concept leads to a factor of 1 that is used. Therefore, application of correction factors is only helpful if the majority of parasites identified in the dataset actually belong to the species for which correction factors are available. The nemabiome approach has so far been applied to investigate the strongyle communities of cattle [17, 22], sheep [21, 23], goats [24], water buffaloes [25], bisons [26], wild ruminants [27, 28], alpacas [29], pigs [30], horses [31–34], zebras [35], and dogs [36]. It has also been shown to allow the identification of species that survived treatment and characterize parasite communities after a FECRT was conducted [37–40]. However, variation between replicates of FECs and also variation between deep sequencing results for the same sample [39] are too high to really allow calculation of FECR for individual species on the basis of a combination of FECs and relative abundance calculated from read counts. The fact that correction factors are only available for some species further complicates the interpretation. Although frequencies of DNA reads in deep amplicon sequencing data do not directly reflect frequencies of species in eggs that are shed or larvae that developed from cultured eggs, the method is currently the most advanced to characterize strongyle communities in hosts [18]. Deep amplicon sequencing targeting the isotype-1 β-tubulin gene and the acr-8 gene has also been used to quantify polymorphisms associated with benzimidazole [35, 41] and levamisole resistance [42], respectively.

Knowledge about species composition of nematode communities will improve, e.g., the knowledge regarding disease ecology, pathogenicity in single- and mixed-species infections, and the contribution of individual species to treatment failure and resistance.

Old World camels are known to react very sensitive to GIN infections, which can trigger different symptoms and diseases depending on the type of parasite [11]. *Haemonchus* spp. cause anemia, weight loss, and general weakness, while *Cooperia* and *Trichostrongylus* species can cause enteritis [12, 13, 43]. A non-specific but common symptom of the disease is diarrhea. If left untreated, this is the main cause of death in OWCs [44]. Owing to their high susceptibility and frequent exposure to GIN, OWCs are regularly treated with anthelmintics [11]. Internationally, products licensed to deworm OWCs contain the benzimidazoles albendazole (ABZ) and fenbendazole (FBZ), the macrocyclic lactones ivermectin (IVM) and doramectin, the imidazothiazole levamisole, and the tetrahydrobromide pyrantel. However, in the European Union, no anthelmintics have been licensed for the treatment of Old and New World camels, and drugs have to be used that have been licensed for the same purpose in ruminants or horses. Veterinarians have to consider if different dose rates need to be used compared with the original species for which the drug was licensed.

Owing to the widespread occurrence of anthelmintic resistance in most grazing domestic animals [45, 46], it is highly recommended to control the efficacy of anthelmintic treatments regularly [47]. The fecal egg count reduction test (FECRT) is still the most widely applied test to evaluate the resistance/susceptibility status of communities of GIN [47]. For a long time, the FECRT was conducted and interpreted according to a guideline of the World Association for Veterinary Parasitology (WAAVP) published by Coles et al. in 1992 [48]. However, this guideline has been recently revised [47], and the criteria to diagnose anthelmintic resistance were completely changed [47, 49]. In addition, multiple statistical methods to calculate confidence or credible intervals for the FECR estimate have been described over the years. The most sophisticated statistical approaches considering different sources of variation have been used in hierarchical Bayesian models, either based on parametric (eggCounts) or non-parametric (bayescount) assumptions for data distribution [49, 50].

The present study aimed to characterize the composition of strongyle communities in OWCs in Germany and find out whether they were dominated by typical OWC GIN species, such as *H. longistipes*, or more by typical ruminant species such as *H. contortus*. In addition, the project aimed to determine the efficacy of standard anthelmintic treatments on German farms keeping OWCs as well as to compare different statistical approaches and interpretation frameworks to conclude which resistance status should be assigned to different datasets. Moreover, the study aimed to determine which strongyle species had the highest probability to survive current treatment practices.

## Methods

### Study design

For this study, farms, zoos, and other establishments in Germany (all summarized as farms in the following) that owned a minimum of eight OWCs were sought. The inclusion criteria were that the farms planned their routine anthelmintic treatment for the entire population between March 2023 and July 2023 and that the animals had not received deworming for at least 8 weeks before the pretreatment sample was taken. Selection of OWC farms was not carried out at random but based on the agreement of farm owners and the above-stated inclusion criteria. A call to recruit club members of an association of OWCs keepers (Altweltkamele e.V.) was initiated. The call provided information about the study and encouraged participation by emphasizing the advantages for the owners/farmers. In addition, individual farm owners were specifically contacted.

### Questionnaire

For the statistical analysis, data from the farms was collected using a questionnaire (Additional file 1: Questionnaire). Data were requested from the owners of the establishment regarding the total number of other animal species kept on the farm and the total number of OWCs, the age and sex composition of the OWC herds, purchases or possible additions per year, stable and pasture times, area changes and mixed grazing with ruminants and new world camelids, the stable and pasture hygiene management, feeding and drinking points, feeding, the supply of additional minerals and salt, the extent to which the OWCs were moved outside of the farm pastures, the regularity of coproscopic diagnosis on the population, as well as deworming criteria and use of anthelmintic drugs, the anthelmintics active compounds used, the treatment dosage, the self-assessment of observed clinical signs of infection in the animals and the self-assessment of deworming measures on the farm. For all animals examined, an individual data sheet was also included with information on age, sex, additional information (pregnant, lactating, and castrated), last deworming, and the origin of the individual OWC.

### Coproscopic examinations

Individual fecal samples were collected from the ground immediately after defecation so that the samples could be assigned to the individual animal and contamination with soil material could be ruled out. Samples were collected using nitrile gloves and stored in individually labeled Ziplock bags. The bags containing the fecal samples were then transported to the laboratory in a fridge box (4 °C). Samples were analyzed on the same day of collection or at a maximum of 1 day later (Additional file 1: Table S1). The deworming of the OWCs on the farms took place as part of routine anthelmintic treatment, and the active ingredient and the dosage were prescribed by the veterinarian of the farm in consultation with the animal owners and not influenced by the authors of the study. In all cases, deworming was conducted before the first grazing of the season so that all farms differed concerning the treatment time point, and accordingly, the collection of the pretreatment sample occurred between April 4th and June 4th, 2023. To investigate the efficacy of anthelmintics, the aim was to compare the individual fecal samples from the animal owners between days 0 and 14 after deworming. The samples were then delivered to the laboratory via express delivery in a Styrofoam box with pre-cooled ice packs. After arrival at the laboratory, samples were stored at 4–8 °C.

The fecal samples were examined for eggs of parasitic nematodes using the quantitative flotation method FLOTAC [51]. Feces (10 g) were weighed and homogenized with 90 ml of tap water. The fecal suspension was then passed through a sieve (0.5–1 mm mesh size), and the filtrate was collected. The filtrate was then mixed with a transfer pipette, and 11 ml were placed in a centrifuge tube, which was then centrifuged at 704 × g for 2 min. The supernatant was decanted. To the remaining sediment in the centrifuge tube, 11 ml of saturated sodium chloride solution (density: 1.2 g/cm^3^) was added, and the sediment was resuspended. The fecal suspension was filled into the two chambers of a FLOTAC unit and centrifuged at 313 × g for 5 min [51]. For quantitative evaluation, both FLOTAC chambers were counted under the microscope (100 × magnification). In this procedure, one egg under the microscope in the FLOTAC unit corresponds to an EPG of 1. The procedure was repeated on individual fecal samples at about 14 days after anthelmintic treatment.

### Isolation of gastrointestinal strongylid eggs

To purify the GIN eggs, the individual fecal samples (that had not been used for the FLOTAC procedure) from the same farm and treatment group (before and after treatment) were mixed in a bucket as described previously [40].

To arrest egg development, the fecal samples were mixed with 1% Lugol’s iodine solution (in a ratio 1 g feces to 2 ml 1% Lugol’s solution (100% Lugol’s stock 250 g KI, 125 g I_2_ dissolved in 250 ml H_2_O) and stored in a cold room at 4–8 °C [52]. The Lugol’s fecal suspension was then repeatedly sieved, washed, and floated to separate the eggs from the other particles. The Lugol’s solution/feces suspension was sieved through a sieve with a mesh width > 500 µm by rinsing with tap water. The flow-through was collected until it became clear. The remaining feces were squeezed out over the sieve and discarded. Then, a 160 µm mesh size sieve was placed on a clean bucket. The collected filtrate from the previous step was sieved, occasionally rinsing the sieve with water to prevent the sieve from clogging. In addition, the fecal material that got stuck in the sieve was rinsed again and then squeezed out and disposed.

A sieve with a 25 µm mesh size was then used, and the remaining filtrate was sieved, rinsing again with water until the flow through was clear. The eggs were collected from the top of the 25 µm sieve. The material collected from the top of the sieve was concentrated by centrifugation at 1956 × g for 5 min. The supernatant was decanted with approximately 10 ml of the liquid remaining above the sediment. The pellet was mixed with saturated NaCl solution to achieve a final volume of 50 ml and centrifuged at 1956 × g for 5 min. The top 15 ml from the centrifuge tubes were pipetted onto a 25 µm mesh size sieve. The sieve was then rinsed with tap water to remove the salt. The remaining egg suspension in the sieve was transferred to a clean centrifuge tube.

For the following density gradient centrifugation, 40, 25, and 10% dilutions of a concentrated sugar solution (60 g sucrose in 40 ml distilled water) were used. Dilutions were stained with 2 drops of different food colors to improve the visibility of borders between density steps. For the density gradient centrifugation, 15 ml of the sugar gradient solutions were layered below each other in conical centrifugation tubes, starting with the lowest density solution. The egg suspension was then carefully pipetted on the top of the step gradient. Gradients were then centrifuged at 1956 × g for 5 min without a break. After centrifugation, the eggs were visible as a thin veil between the 25% and 10% dilution steps. The upper part of the gradient was sieved through a 25 µm sieve, washed with tap water to remove the sugar before eggs were recovered from the top of the sieve and transferred to 15 ml tubes, which were centrifuged at 1956 × g for 5 min. After careful decantation and resuspension of the eggs in a small volume of water, eggs were counted by transferring 2 × 10 µl of the suspension onto a microscope slide and examination at 100 × magnification to calculate the total amount of isolated eggs. Finally, egg suspensions were stored at −20 °C until further use.

### DNA isolation

To isolate the genomic DNA of the GINs, the NucleoSpin^®^ 8 Soil Kit (Macherey–Nagel, Düren, Germany) was used for the isolation of nematode DNA from eggs collected from fecal samples [40, 52, 53]. The kit contains tubes with beads that were used in a “bead-beating” process to break the eggshells. The isolation followed the manufacturer’s instructions using the Lysis Buffer SL1, and DNA was eluted with 50 µl Buffer SE. DNA was stored at −20 °C.

### Nemabiome library preparation by two-step PCRs

The ITS-2 fragment of strongyle rRNA genes was amplified using modified NC1/NC2 primers [19]. The primers contained zero to three additional random bases (N) between the NC1/NC2 sequences and the Illumina adapters (Additional file 1: Table S2). These additional random bases lead to a scattering of the signal to allow the proper calculation of error correction profiles in the primer region, in which all molecules are identical [17, 22]. The PCR reaction was carried out using the reaction mixture described by Avramenko et al. [17] using details according to Krücken et al. [40].

The first PCR was performed using an initial denaturation at 95 °C for 2 min, 30 cycles with 95 °C for 20 s, 62 °C for 15 s, and 71 °C for 15 s, followed by a final extension at 72 °C for 2 min. Water instead of template DNA was used for the negative controls. For the positive controls, *Oesophagostomum dentatum* plasmid DNA (10^5^ copies/reaction) was used.

### Deep-amplicon-sequencing

PCR products were purified using AmpureXP beads (Beckman Coulter GmbH, Krefeld, Germany) according to the manufacturer’s instructions. The final elution volume was 40 μL in a 10 mM Tris–HCl buffer (pH 8.0) to eliminate primer dimers and protein residues. The purified DNA was then quantified using the Qubit dsDNA HS Assay Kit (Thermo Fisher Scientific, Darmstadt, Germany) on a Qubit 4 Fluorometer (Thermo Fisher Scientific, Darmstadt, Germany). Purified PCR products (10–20 ng) were used to add Illumina adapters. Index PCRs were performed using the Integrated DNA Technologies (IDT) Kit for the Illumina DNA/RNA UD Index Set (Illumina, San Diego, CA, USA) and the KAPA HiFi HotStart Ready Mix (Roche Molecular Systems, Pleasanton, CA, USA). The purified PCR product (3 µl) was mixed with 12.5 µl Kapa HiFi Ready Mix, 1.5 µl dual-index primers, and 8 µl PCR-grade water. The amplification conditions for the Kapa HiFi Ready Mix in a final reaction volume of 25 µl were as follows: first denaturation at 98 °C for 45 s, followed by 7 cycles of denaturation at 98 °C for 20 s, annealing at 63 °C for 20 s, and elongation at 72 °C for 20 s, and a final elongation at 72 °C for 120 s. After the index PCR, the PCR products were purified again with AmpureXP beads according to the manufacturer’s instructions, using an elution volume of 25 μL of 10 mM Tris–HCl buffer (pH 8.0). Final libraries were quantified using the Qubit dsDNA HS Assay Kit and diluted to 4 nM in 10 mM Tris–HCl buffer before pooling. The diluted libraries were pooled in equimolar amounts, denatured, and finally further diluted according to the manufacturer’s protocol (Illumina). The sample pool was sequenced on a MiSeq benchtop sequencer (V3, 2 × 300 bp, Illumina). It was aimed to obtain a minimum of 20,000 paired-end reads per sample.

### Statistical and bioinformatic analyses

Descriptive statistics were performed in Microsoft Excel. The Wilcoxon signed-rank test was used to compare the abundance of parasites in paired samples using GraphPad Prism 5.03. Differences in percentages between groups were analyzed using the mid-p exact test with the tab2by2.test() function from the epitools 0.5–10.1 package in R 4.2.3. The 95% confidence intervals (CI) for the prevalence on a farm were calculated as exact hypergeometric CI using Sprop() from the samplingbook 1.2.4 package taking advantage of the fact that the total number of animals on the farms was known. The binom.wilson() function from epitools was used to calculate 95% CI for farm prevalences, assuming an indefinite number of OWC farms.

The FECR with 95% credible intervals (CrI) was calculated using the package eggCounts 2.4 in R and in parallel with bayescount 1.1.0 implemented as an online version on https://www.fecrt.com/ (last accessed: 30 August 2024). Both statistical models are based on Bayesian statistics and use Markov-Chain-Monte-Carlo models and samples generated from them to determine the CrI for FECR. However, they differ in terms of the mathematical models used for the distribution of EPG values and the modeling of errors in the measurements with eggCounts using a parametric and bayescount, a non-parametric model. The eggCounts package was used to calculate paired samples without zero inflation and without individual efficiency [50]. The values were then classified into three categories according to Denwood et al. resistant, susceptible, and inconclusive, applying the cut-off and the grey zone as defined in the research protocol for ruminants in the WAAVP guideline [47, 49]. The results of the classification into the categories resistant, susceptible, and inconclusive using the two methods were compared in R regarding interrater reliability using Cohen’s Kappa. The CohenKappa() function from the DescTools 0.99.48 package was used for this purpose.

The output of the Illumina Sequencer was demultiplexed, and primers were removed using cutadapt [54] as described previously [40]. The dada2 pipeline [55] is described in detail on the Nemabiome website (https://www.nemabiome.ca/; last accessed: 23 February 2024) [56, 57]. The reads were filtered and trimmed to allow only reads with a maximum expected number of errors of two for the forward read and five for the reverse read. The reads were shortened so that a maximum of two errors could be expected per read. The error profiles of the data set were used to train dada2, and then error correction was carried out using dada2’s “denoising” function. Then forward and reverse “reads” were merged as detailed by Avramenko et al. [17] and on the nemabiome.ca webpage (last visited 31 May 2025). The IdTaxa function from the DECIPHER 2.22.0 package [58] was used to assign the sequences to specific taxa, applying 100 bootstrapped pseudo-replicates and a clamp of 60%. Version 1.4 of the nemabiome database [59] was used for species identification. The frequencies of all species were calculated for each sample from the reads, applying species-specific correction factors [17, 21, 22].

## Results

### Study population

**Fig. 1:**
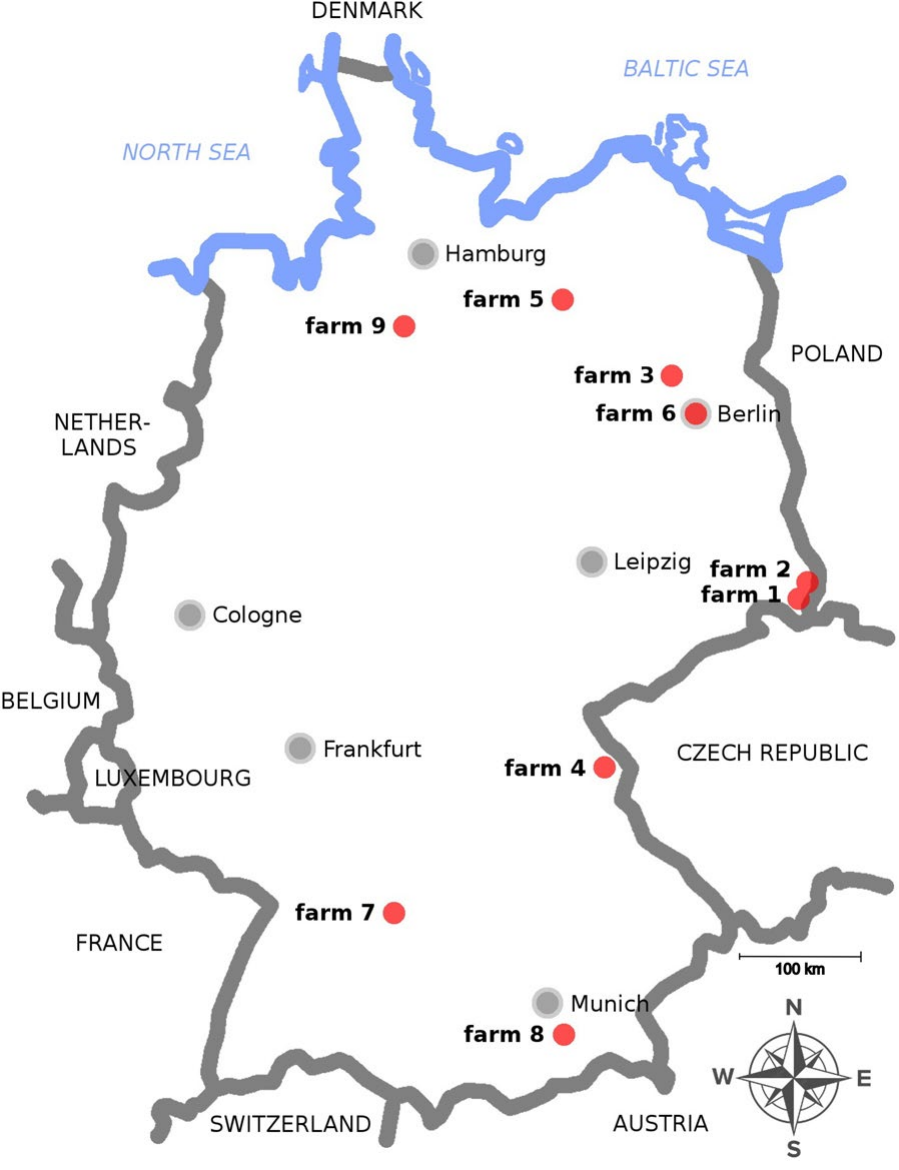
Overview map of the Federal Republic of Germany with the locations of the sampled farms (B1–B9)

Data collection and sampling for the present study were conducted between 4 April 2023 and 4 June 2023. On eight of the farms, animals were dewormed before turnout to the pasture, while on farm B6, the animals had year-round pasture access. In total, data were collected from nine farms (Fig. 1), and samples from 107 OWCs aged 0–20 years in Germany were included (Fig. 1). Two farms did not meet the originally established inclusion criteria since farm B6 did not carry out any anthelmintic treatment and B1 only kept seven OWCs (inclusion criterion ≥ 8 animals). Owing to the small number of available and willing to participate OWC farms in Germany, it was ad hoc decided that the two farms were not excluded. The total population of all OWCs on the farms was divided into 63.5% (108/170) *C. bactrianus*, 28.8% (49/170) *C. dromedarius,* and 7.7% (13/170) hybrids. Six farms kept only Bactrian camels, while three farms kept mixed groups (1 × Bactrians and dromedaries, 1 × Bactrians and hybrids, 1 × Bactrians, dromedaries, and hybrids). Two camel groups were kept in zoos (22.2%), one farm (11.1%) was a commercial camel breeder, and six farms (66.7%) kept their animals for the recreational sector. The animals included were represented by 92 Bactrian camels (86.0%), 7 dromedaries (6.5%), and 8 hybrids (7.5%). The GIN EPGs of the 107 animals were recorded by coproscopic diagnostics. The collection of the fecal samples before the anthelmintic treatment (pre) was carried out by JB, except for B6 (carried out by the animal keepers). On three farms, the number of individual fecal samples was less than eight. Farm B1 held only seven OWCs, and for two other farms, there were difficulties collecting samples, so that for B5, only seven, and for B6, only six individual samples were available. Furthermore, farm B6 decided not to carry out any anthelmintic treatment, and farm B2 was excluded from the FECRT analysis because the animals on this farm had too low EPG values (mean EPG 1.8) before treatment. Thus, analyses of FECR data were conducted on seven farms with a total of 100 animals participating in the study of the efficacy of anthelmintics.

The post-treatment fecal samples were collected by the farm owners according to detailed instructions. On some farms, post-treatment samples were not collected on day 14 but between days 13 and 21 (Additional file 1: Table S1). The delivery time was between one and 5 days (mean 2.3 days).

The farm prevalence of strongyle egg shedding before treatment was generally high and ranged from 50 to 100% (Table 1). According to the results of the mid-p exact test, treatment led to a significant reduction of prevalence in only two out of eight farms (B2, B9). The 95% CIs for prevalence were not overlapping for three farms (B2, B9, and, in addition, B1). In contrast to the mid-p exact test, the 95% CIs consider the fact that on many farms, the majority or even all animals were included, and the population size was not indefinite. For data pooled from all farms, there was a significant decrease in prevalence after treatment (Table 1).

**Table 1:** Prevalence of strongyle eggs in fecal sample pre and post treatment

Farm	N^a^	Pretreatment		Post treatment		*P*-value^c^
		Prevalence [%]	95% CI^b^	Prevalence [%]	95% CI^b^	
B1	7	100	100–100	57.1	57.1–57.1	0.096
B2	12	50.0	50.0–50.0	0	0–0	**0.007**
B3	14	78.6	73.3–80.0	78.6	73.3–80.0	1
B4	8	87.5	87.5–87.5	87.5	87.5–87.5	1
B5	7	100	87.5–100	100	87.5–100	1
B6	7	87.5	50.0–93.8	n.a.^d^	n.a.^d^	n.a.^d^
B7	20	94.4	85.0–95.0	94.4	85.0–95.0	1
B8	15	100	100–100	100	100–100	1
B9	19	94.7	76.8–98.6	52.6	31.9–73.9	**0.004**
Total	170	87.9	82.9–91.8	71.0	64.1–77.1	**0.003**

^a^Total number of animals investigated. ^b^Exact hypergeometric 95% confidence interval. ^c^Mid-p exact test. ^d^Not available. Significant differences (P < 0.05) are highlighted by bold *P* values

**Fig. 2:**
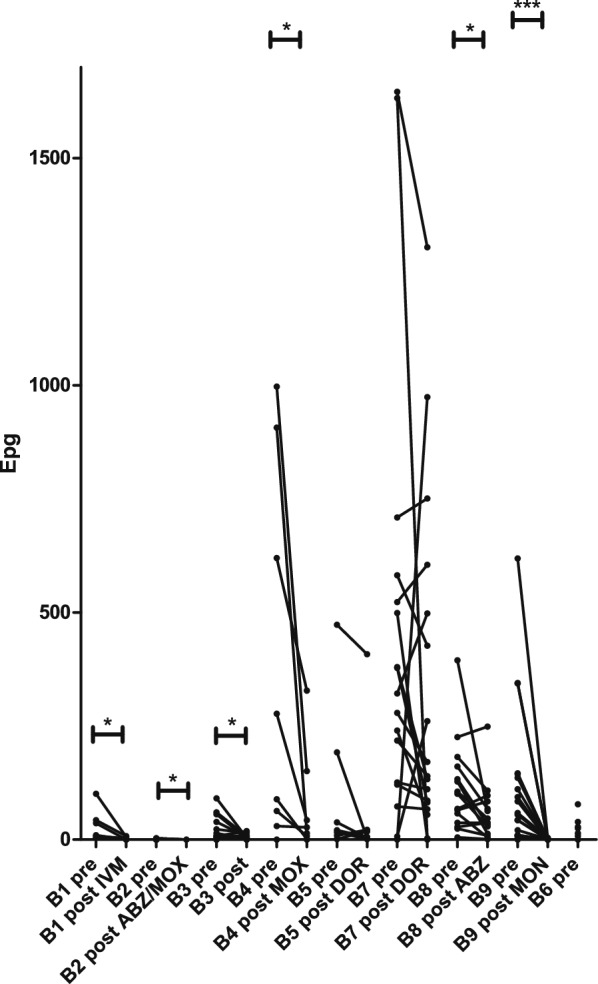
Comparison of eggs per gram feces (EPGs) for individual Old World camels pre- and post-treatment with different anthelmintics. Each data point represents an individual animal, and values from pre- and post-treatment samples are connected by a line. Samples are named using the farm code (B1–B9), the sampling time (pre-, post-treatment), and the anthelmintic used (IVM, ivermectin; MOX, moxidectin; FBZ, fenbendazole; ABZ, albendazole; DOR, doramectin; MON, monepantel). Farm B6 did not perform any anthelmintic treatment after the first sample collection. The Wilcoxon sign-rank test was used to compare EPGs pre- and post-treatment. *, *P* < 0.05; ***, *P* < 0.001

The strongyle egg shedding abundance varied widely between farms, with very low EPG values on farm B2 and very high values on farms B7 and B4 (Fig. 2). The maximum EPG was 1646, and 20.8% of the OWCs showed an EPG > 200. Treatment led to a significant decrease in egg-shedding abundance on six out of eight farms (not on B5 and B7, the latter being the farm with the highest pretreatment EPG values).

### Fecal egg count reduction test

To calculate the FECR, data from seven farms with a total of 88 individual OWCs were analyzed. Farm B2 was excluded since the pretreatment EPG values were too low to obtain reliable FECR estimates. Table 2 shows that the classification of the results for eggCounts and bayescount were identical. Likewise, it was irrelevant for the classification whether the revised guideline with the assignment based on upper and lower 90% credible limits (CrLs) or the original guideline with criteria based on the estimated FECR and the lower 95% CrL was applied. With each of the three methods used, results of the FECRT for 85.7% (6/7) of the farms were classified as resistant, and for one farm, was classified as susceptible. This resulted in an interrater reliability determined as Cohen’s κ = 1. Farm B7 (DOR treatment) had the lowest FECR (26.6%) and the lowest 90% CrL value compared with the other farms (Table 2). In contrast, B9 (MON treatment) had the highest FECR (99.3%) and the highest lower 90% CrL (Table 2).

**Table 2:** Statistical analysis of the fecal egg count reduction test and classification of the efficacy of the anthelmintic treatments used for the individual farms comparing eggsCounts (with 90% and 95% credible limits (CrLs)) and bayescount (with 90% CrLs) statistical approaches

Farm	B1	B3	B4	B5	B7	B8	B9
Medication	Bimectin	Albendazole 10%	Cydectin 0.1%	Dectomax	Dectomax	Albendazole 10%	Zolvix
Target host species	Horses	Ruminants	Sheep	Ruminants	Ruminants	Ruminants	Sheep
Application route	Oral	Oral	Subcutanous	Subcutanous	Subcutanous	Oral	Oral
Drug^a^	IVM	ABZ	MOX	DOR	DOR	ABZ	MON
Drug class^b^	ML	BZ	ML	ML	ML	BZ	AAD
**Statistical method**							
**eggCounts**							
FECR (%)	90.8	76.4	80.9	38.4	26.6	48.5	99.3
90% CrL low	87.6	71.0	79.3	31.7	24.4	45.0	99.0
90% CrL up	94.8	80.9	82.3	44.0	28.6	52.0	99.6
Classification	**Resistant**	**Resistant**	**Resistant**	**Resistant**	**Resistant**	**Resistant**	**Susceptible**
95% CrL low	86.3	70.5	78.9	30.6	23.8	44.2	99.0
95% CrL up	95.0	82.3	82.4	45.4	28.8	52.4	99.6
Classification	**Resistant**	**Resistant**	**Resistant**	**Resistant**	**Resistant**	**Resistant**	**Susceptible**
**Bayescount method**	Delta	Delta	Delta	Delta	Delta	Delta	Delta
90% CrL low (%)	80.2	57.2	55.3	-92.5	-24.1	15.6	98.8
90% CrL up (%)	98.2	90.9	96.4	97.7	65.7	74.6	99.7
Classification	**Resistant**	**Resistant**	**Resistant**	**Resistant**	**Resistant**	**Resistant**	**Susceptible**

^a^IVM, ivermectin; ABZ, albendazole; MOX, moxidection; DOR. Doramectin; MON, monepantel

^b^ML, macrocyclic lactone; BZ, benzimidazole; AAD, aminoacetonitrile derivative

### Analysis of treatment protocols

Of the farms examined, 88.9% (8/9) had carried out deworming. Although farm B2 was excluded from the FECR analysis owing to the low egg excretion in the pretreatment samples, it was included for the analysis if the applied dosage was appropriate with both drug classes that were applied in combination. According to the data recorded with the questionnaire (Additional file 2: Table S3), three farms weighed the animals before treatment (B2, B6, B8), while the others only estimated the animal weights. To determine if reasonable doses of anthelmintics were used to treat the animals, two different aspects need to be considered. As the first aspect, it must be questioned whether the weight of the animals was determined correctly since, with an imprecise weight determination, dose determination will also be inaccurate, and under- and/or overdosing might be very common. In fact, only three out of eight farms that were treated with anthelmintics used a scale to determine the weight of the animals, while on the other five farms, the animal weight was estimated. This indicates that dosages might often be wrong due to missing data on the correct weight of the animals. The second aspect regards the fact that all drugs were given off-label since there are no licensed drugs available for OWCs in Germany and the EU. On the other hand, ABZ, FBZ, IVM, and DOR are at least licensed for OWCs in other countries. In the current study, products that were licensed for ruminants and horses had been used for the treatment of OWCs. This also means that there is no dosage for OWCs stated in the leaflet, and veterinarians have to rely on other sources of information to determine the optimal dosage. A common source of information for German veterinarians regarding drug use is Veterinary Information Service for Drug Use, Toxicology and Drug Law (VETIDATA; www.vetidata.de, last accessed: 10 September 2024). Dosages recommended by VETIDATA for common ruminant and camelid species are shown in Table 3, and values for OWCs stated in VETIDATA were given according to Fowler [60]. In addition, Table 3 provides doses suggested for OWCs by Faye et al. [1]. Comparison of these suggested dosages with the dosages that were actually applied–assuming that the weight was determined correctly–were at least as high or considerably higher than the suggested doses, with the exception of farm B7, where animals received between 80 and 180% of the recommended dosage of doramectin. These data show that underdosing due to applying a lower dose is probably not a widespread problem.

**Table 3:** Calculation of the anthelmintic dosage used by the farms per active substance class in accordance with the approval for sheep and the Camelidae recommendation

Drug class	Drug	Application route	Target species	Recommended dosage (mg/kg) VETIDATA	Recommended dosage (mg/kg) [1]	Farm	WD^a^	Applied dose (mg/kg)^b^
ML	IVM	Oral	Sheep	0.2				
			Cattle	0.2				
			Horses	0.2				
			Goat	0.2–0.4				
			Alpaca	0.2–0.6				
			Bactrian	0.2	0.2	B1	E	0.36–0.6
			Dromedary	0.2	0.2			
ML	MOX	Oral	Sheep	0.2				
			Cattle	0.2				
			Horses	0.4				
			Goat	0.4				
			Alpaca	0.4				
			Bactrian	0.4	0.4	B2 B4	S S	0.4–0.42^c^ 0.4
			Dromedary	0.4	0.2			
ML	DOR	Subcut-aneous	Sheep	0.2				
			Cattle	0.2				
			Horses	n.a				
			Goat	0.2				
			Alpaca	0.2–0.44				
			Bactrian	0.2	0.2	B5 B7	E E	0.24 0.16–0.36
			Dromedary	0.2	0.2			
BZ	ABZ	Oral	Sheep	3–5				
			Cattle	7.5				
			Horses	5				
			Goat	3.8–10				
			Alpaca	10–15				
			Bactrian	5–7.5	5–7.5	B3 B8	E S	10.2–12 10–15.5
			Dromedary	5–7.5	7–10			
BZ	FBZ	Oral	Sheep	5				
			Cattle	5–7.5				
			Horses	10				
			Goat	5				
			Alpaca	5–15				
			Bactrian	n.a	5–7 (for 3 days)	B2	S	9.5–10.5^c^
			Dromedary	n.a	5–7.5			
AAD	MON	Oral	Sheep	2.5				
			Cattle	n.a				
			Horses	n.a				
			Goat	7.75				
			Alpaca	n.a				
			Bactrian	n.a		B9	E	4.25–5
			Dromedary	n.a				

^a^Weight determination: S, scale; E, estimated

^b^Same dose was applied to Bactrian camels and dromedaries (and hybrids) if both species were present

^c^Used as combination treatment with MOX and FBZ

ML, macrocyclic lactone; BZ, benzimidazole, AAD aminoacetonitrile derivatives; IVM, ivermectin; MOX, moxidectin; DOR, doramectin; ABZ, albendazole; FBZ, fenbendazole

### Strongyle egg isolation

The pretreatment samples from all nine farms and the post-treatment samples from six farms were used for GIN egg isolation. Two (B2, B9) of the nine farms were excluded from egg isolation from post-treatment samples because the EPG data for the entire follow-up population was too low. Furthermore, one farm (B6) did not carry out deworming, so no post-treatment data was available.

For egg isolation, all samples from the same farm and time point were pooled. Isolation of the GIN eggs was successful for 14/15 samples, and the number of isolated eggs is shown in Additional file 1: Table S4. For farm B2 before treatment, no eggs were detected microscopically. Since only a small aliquot of the sample was used for counting, the sample was nevertheless included for DNA isolation and PCR since it was possible that it contained a few eggs and/or free nematode DNA.

### ITS-2 PCR and deep-amplicon sequencing

**Fig. 3:**
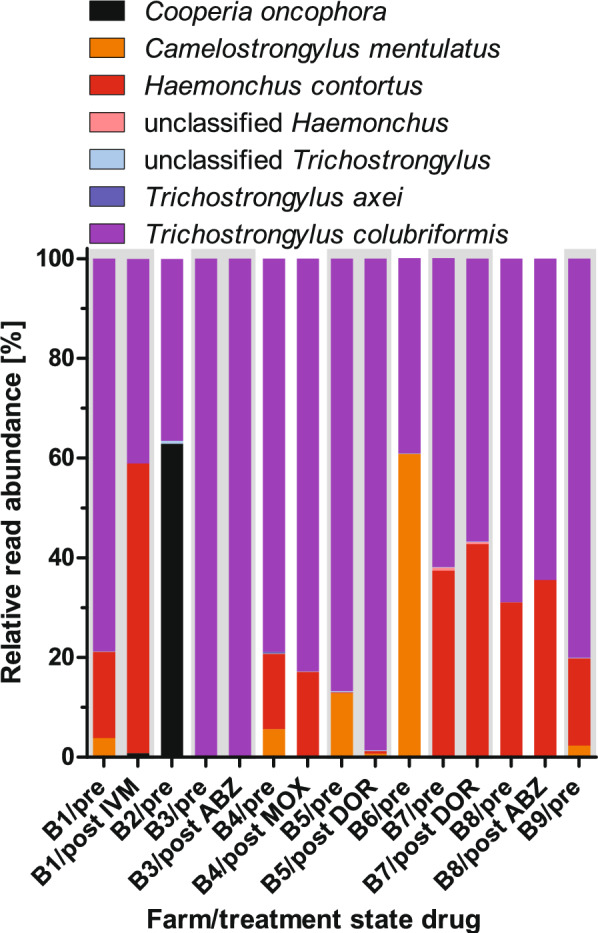
Relative read abundance for different strongyle species obtained from isolated eggs on farms B1 to B9 before (pre) and after (post) treatment. Grey and white background colors were used to separate different farms. For farms B2, B6, and B9, no post-treatment samples were available since the animals were not treated (B6) or the number of eggs post-treatment was so low that no eggs could be isolated

For each farm, one aliquot of eggs was used for DNA isolation and ITS-2 PCR. The pretreatment sample for farm B2 was also included, although no eggs were detected microscopically. PCRs for all samples were positive, including the B2 pretreatment sample. Thus, nemabiome sequencing was performed on a total of 15 samples (9 pre- and 6 post-treatment). Statistics of raw reads obtained per sample and numbers of reads lost during the different bioinformatic steps are provided in Additional File 1: Table S5. The raw R output of the nemabiome pipeline is provided in Additional file 3: Nemabiome R console in- and output. The farm prevalence and the frequency of reads for different strongyle species for each sample pre- and post-treatment are shown in Table 4 and Fig. 3. *Trichostrongylus colubriformis* was the most prevalent nematode occurring in all samples before and after treatment. In 7/9 pretreatment and 5/6 post-treatment samples, it was also the most abundant parasite according to read counts from egg samples. In all other samples, *T. colubriformis* was the second most abundant parasite. Unclassified *Trichostrongylus* were found in many samples before and after treatment but at a very low read frequency (range 0–0.6), while *T. axei* was detected in only a single pretreatment sample (0.3% of all reads). Since differences in ITS-2 sequences are rather small between species of the same genus, and in some cases the ITS-2 is not even suitable to discriminate between species [61, 62].

**Table 4:** Farm prevalence with 95% confidence intervals (CIs) for strongyle species on Old World camel farms in Germany

Species	Pretreatment		Post treatment		*P* value^b^
	Prevalence [%]	95% CI^a^	Prevalence [%]	95% CI^a^	
*Trichostrongylus colubriformis*	100	70.1–100	100	61.0–100	1
*Trichostrongylus axei*	11.1	2.0–43.5	0	0–39.3	0.600
Unclassified *Trichostrongylus*	100	70.1–100	66.7	30.0–90.3	0.143
*Haemonchus contortus*	66.7	35.4–87.9	83.3	43.6–97.0	0.554
Unclassified *Haemonchus*	11.1	2.0–43.5	16.7	3.0–56.4	0.750
*Cooperia oncophora*	22.2	6.3–54.7	16.7	3.0–56.4	0.844
*Camelostrongylus mentulatus*	66.7	35.4–87.9	16.7	3.0–56.4	0.369

^a^95% confidence interval

^b^Mid-p exact test

The second most prevalent parasite was *H. contortus*, which was detected in two-thirds of the pretreatment samples, and prevalence even increased to 83.3% in post-treatment samples. In five out of nine pretreatment samples, *H. contortus* was the second most abundant parasite, while in post-treatment samples, it was the most abundant parasite in one and the second most abundant parasite in three of the six samples. In addition to *H. contortus*, a small fraction of the reads was assigned to unclassified *Haemonchus* on one farm in both pre- and post-treatment samples. Like *H. contortus*, *C. mentulatus*, a member of the family Trichostrongylidae, occurred on 66.7% of the farms before treatment but was found on only 1/6 farms after treatment. The relative abundance of *C. mentulatus* readings was usually low to moderate (< 13%), but on one farm, it was the most abundant parasite. Finally, *C. oncophora* was found on 22.2% of the farms before and in 16.7% of post-treatment samples. In one of the pretreatment samples, *C. oncophora* was the most abundant parasite, while read frequencies were below 1% for the other two positive samples.

The effects of treatments are described for drug classes individually. Albendazole alone was used on farms B3 and B8, resulting in FECRs of 76.4 and 48.5%, respectively (Table 2). On B3, virtually only *T. colubriformis* were found in both pre- and post-treatment samples. On farm B8 the dominant species was also *T. colubriformis*, but there were also about 31% *H. contortus* (Fig. 3). After treatment, the relative abundance of *H. contortus* increased slightly to approximately 36%, while the frequency of *T. colubriformis* reads was reduced accordingly (Fig. 3). This suggests that the *T. colubriformis* populations on B3 and B8 and the *H. contortus* population on B8 were resistant to ABZ. Macrocyclic lactones were used on five farms, i.e., IVM on B1 (FECR 90.8%), DOR on B5 (FECR 38.4%) and B7 (FECR 26.6%), and MOX on B4 (FECR 80.9%) (Table 2). On B1, the relative abundance of *H. contortus* reads increased post-treatment from about 17–58%, suggesting that this population is very resistant. The relative abundance of *T. colubriformis* was approximately halved by IVM treatment (from 79 to 41%), suggesting that this parasite is at least partially resistant but less so than *H. contortus* in the same community (Fig. 3). In contrast, the *C. mentulatus* (3.8% before treatment) were completely eliminated by IVM, showing that this species was susceptible. Unexpectedly, there was a low frequency of *C. oncophora* (0.8%) that was only detected in the post-treatment sample. Treatment with DOR led to an increase in the relative abundance of *T. colubriformis* from 87 to 99% on farm B5, while the relative abundance of this parasite slightly decreased after DOR treatment from 62 to 57% on B7 (Fig. 3). Considering the low FECR on these farms, both *T. colubriformis* populations can be considered resistant. The remarkable difference between both farms is the high frequency of *H. contortus* on B7, which increased from 37 to 43% following DOR treatment (Fig. 3). On farm B5 the relative abundance of *H. contortus* was only 0.2% before treatment and then increased to only 0.5% (Fig. 3). Obviously, both *H. contortus* populations were DOR resistant and on B7 *H. contortus* was able to increase its relative abundance in comparison to *T. colubriformis* upon DOR treatment suggesting that *H. contortus* shows a stronger resistance phenotype. The *C. mentulatus* population on B5 was almost completely eliminated by DOR treatment suggesting that it is still susceptible to this drug (Fig. 3). On farm B4, the relative abundances of *H. contortus* and *T. colubriformis* slightly increased upon MOX treatment while the small fraction of *C. mentulatus* was only detectable before treatment showing the presence of MOX resistant *H. contortus* and *T. colubriformis* and a MOX susceptible *C. mentulatus* population on farm B4 (Fig. 3). On farm B9, populations of *T. colubriformis*, *H. contortus* and *C. mentulatus* were detected (Fig. 3) and apparently fully susceptible to treatment with MON according to the results of the FECRT (Table 2).

## Discussion

Infections with GIN and reduced efficacy of anthelmintics lead to significant health problems in domestic ruminants worldwide [63], and this is also the case in Germany [40, 64, 65]. Anthelmintic resistance represents a particular challenge in animal husbandry, with not only resistance against all available drug classes occurring and often being widespread but also a high frequency of multi-drug-resistant parasite communities and populations [63]. There are still significant gaps in knowledge about anthelmintic resistance in domestic ruminants [63]. This applies even more to animal species such as OWCs that are not part of traditional animal husbandry in many countries with industrialized livestock production, such as Germany.

To achieve a high statistical power, it was initially planned to only include farms that keep at least eight OWCs. However, in the end some farms were included for which the number of included animals was below eight. Reasons for a lower number of animals included than animals present on the farm were that not all animals were available at the study day or that it was not possible to obtain a fecal sample that could be unequivocally assigned to an individual animal. Since rectal sampling of feces was avoided, animals on some farms had different owners, who did not all agree to participate, and not all animals defecated in the observing period, the number of animals that actually participated in the study was on some farms lower than initially expected.

The present study showed that infections with GIN can potentially cause a health risk for OWCs on German farms, which was in particular indicated by the high prevalence (overall prevalence 87.9%), while EPGs were mostly moderate (mean EPGs 174.0, median 59.0). Although median EPG values were moderate, there were strong differences in the intensity of strongyle infection between individual animals, and 20.8% of the camels had an EPG > 200, and the maximum was an EPG of 1646, indicating that a substantial number of animals suffered from a very intense infection, and further studies addressing clinical alterations caused by high worm burdens should be conducted. In this context, it must be further taken into account that most pretreatment samples were collected before the animals had pasture access after the winter break, with the exception of farm B6, which had year-round pasture access. Thus, it must be assumed that the prevalence and intensity of infection will further increase during the grazing season compared with these values obtained in spring.

Eight of the nine farms carried out strategic anthelmintic treatment, i.e., deworming of the entire herd at certain time points of the year, such as turnout onto pasture, while farm B6 did not perform any deworming in the last years. A significant reduction in GIN prevalence after treatment was only observed on farms B2 and B9. Farm B2 was the farm with the lowest pretreatment prevalence and strongyle egg shedding abundance and the only farm that previously used a combination of two anthelmintics from different drug classes to treat the animals. On farm B9, MON was applied, and the nematode community was found to be susceptible to this rather new anthelmintic [66]. For all other farms, the GIN prevalence did not decrease, or the decrease was not significant. Regarding egg-shedding abundance, the effect of anthelmintic treatment was better since on five out of seven farms, a significant reduction of EPGs due to treatment was found, with the two exceptions, i.e., farms B5 and B7, which were both treated with DOR. On farms B5 and B7, the OWCs were treated with DOR and only resampled on days 19 and 21 (Additional file 1: Table S1), which is longer than the recommended time of 14–17 days [47]. However, the leaflet coming with the Dectomax drug states that it protects against *C. oncophora* for at least 21 days, and against *Ostertagia ostertagi* for at least 28 days. The Doramec (licensed for camels in other countries) leaflet states protection against *H. contortus* and *C. oncophora* for at least 14 days, against *O. ostertagi* for 21 days, and against *Cooperia punctata* for 28 days. Considering an additional prepatency period of at least 2 weeks and the fact that doramectin is active against hypobiotic stages of most strongyle nematodes, the risk that the later sampling points have a strong effect on the results and interpretation of the FECRT was considered to be very low. The decrease in EPGs after treatment likely masks the presence of anthelmintic resistance on many farms, which might not be recognized by farmers since they do not perform quantitative coproscopic analyses and a formal FECRT with statistical analysis but observe clinical improvement of the animals owing to partial reduction in worm burden.

The results of the FECRT, for which meaningful results were obtained for seven farms in the present study, show that on all but one farm, the strongyle community was classified as anthelmintic resistant. However, the FECRT has several limitations in the present study, and the conclusions drawn from the test should be considered with care. First, on four out of the six farms for which the strongyle communities were classified as resistant, the weight of the animals was not determined using a scale but it was estimated. Therefore, underdosing of the animals on these farms cannot be excluded. Second, none of the products used for deworming was licensed for OWCs, and it is, therefore, unclear if the pharmacokinetics are similar to those in the target host species. However, anthelmintic products that contain ABZ, FBZ, IVM, and DOR are marketed in other geographic regions, such as Arabic countries. Third, no data are available regarding the efficacy of the drugs in OWCs when susceptible parasites are treated. Therefore, the thresholds for the expected efficacy of 99% and an acceptable grey zone of 95–99% were chosen according to the commonly used values for ruminants applying the research protocol for the FECRT [47]. However, for the off-label use of an anthelmintic in a different host species, this might be too stringent. Fourth, on most farms, drugs were applied by the farmer and not a trained veterinarian, and oral application of drugs in camels can be difficult due to the size and strength of the animals and a high risk that drugs are spat out [11]. Owing to all these limitations, it might be better to state that the results of the FECRT show an unusually low treatment efficacy, which might, but does not necessarily, be due to anthelmintic resistance.

Despite these limitations, there are several arguments that the observed low treatment efficacies are not only due to problems associated with the treatment of OWCs using non-licensed drugs but also due to parasite-intrinsic properties. First, all samples were unequivocally classified as “resistant” no matter whether the original guideline or the revised guideline was applied or if eggCounts or the bayescount methods were applied. Second, the lower 90% CrL was in the range of 28.6–94.8%, which is considerably below the expected efficacy of 99%, and for all except one farm, the upper 90% CrL was even more than 12% below the lower end of the grey zone. Third, the low efficacy was not due to a few animals on a farm that did not respond to treatment while all others showed high efficacy. Fourth, strongyle communities were also classified as “resistant” if injectable drugs were used. The latter two points rule out that problems with drug application were the major reason for the observed low efficacy. Fifth, the nemabiome data clearly show that treatment was able to eliminate some parasite species (*C. mentulatus*, *C. oncophora*), but not others (*H. contortus*, *T. colubriformis*). This clearly shows that the drugs were not severely underdosed and were able to eliminate at least some species of strongyle GIN. Sixth, the strongyle species that showed a poor response to anthelmintic treatment in the present study were also found to be highly multi-drug resistant on sheep farms in Germany [40], suggesting that resistant parasites were introduced from sheep into camel farms.

The doses recommended in the literature for Bactrian camels and dromedaries are typically not as high as for alpacas but sometimes higher than for sheep and cattle (see Table 3 for details). For alpacas, anthelmintic resistance has already been described in Germany [67, 68].

Apparently, the animal owners and the veterinarians were also aware of the alpaca situation, where, similar to goats, higher doses are required than for sheep and cattle [69]. The doses veterinarians and owners aimed to apply were higher, sometimes two to three times higher than those recommended for Old World camels in the literature. Thus, if underdosing occurred, it was not because owners or veterinarians were not aware that OWC might require a higher dose. However, this does not mean that the situation was the same a decade ago, and previous underdosing might have resulted in resistance nowadays. Probably the more important problem that might lead to underdosing is the fact that the weight of the OWC was estimated on many farms. This is likely to be associated with a high error, which could lead to underdosing.

In New World camelids, in particular in alpacas, it has been shown frequently in recent years that higher dosages of anthelmintics are required and that underdosing is a frequent problem [70, 71]. The situation for OWCs is less clear since recommended doses are often between ruminants and alpacas. Further pharmacological research and evaluation of FECRT data will be required to establish scientifically agreed dosages for OWCs.

To avoid or slow down the selection of anthelmintic resistance, a targeted selective treatment strategy is frequently recommended [72]. This means that only individual animals are treated, and treatment decisions are based on EPG values and poor health parameters associated with GIN infections (e.g., diarrhea, anemia, low body condition score). For instance, the Thünen Institute (German Federal Research Institute for Rural Areas, Forests and Fisheries) recommends for farmers in Germany to treat cattle using a targeted selective treatment scheme and suggests a cut-off for treatment on the basis of an EPG of > 100 [73]. For horses, a cut-off of 200 EPG is often recommended [74–76]. However, there are no evidence-based recommendations for treatment decisions for OWCs available.

We were able to obtain nemabiome data for all nine farms pretreatment and for six out of eight farms post-treatment. Although the number of isolated eggs for farm B2 was estimated to be zero, the PCR was positive, which is probably true owing to underestimation of the number of eggs since only a small aliquot of the sample was counted and/or the presence of free DNA released from destroyed eggs in the sample. The nemabiome data obtained in the present study are, to the knowledge of the authors, the first data on strongyle GIN of OWCs published so far. However, the climate conditions and vegetation that OWCs are exposed to in Germany are very different from the mostly arid or semi-arid habitats where OWCs were traditionally bred, such as central Asia for Bactrian and Arabian countries for dromedary camels. Differences in diet and climatic conditions are expected to have diverse effects on animal health [77, 78], including susceptibility to infectious diseases [11]. Indeed, the strongyle nematode species did not include any of the species occurring exclusively in OWCs listed above in the background section. Remarkably, there was also no evidence that the OWCs-preferring *Haemonchus* species *H. longistipes* was present in any of the samples. In contrast, the species *H. contortus*, the most important and pathogenic strongyle parasite of small ruminants, was widely distributed. Thus, the strongyle communities consisted of some of the most abundant parasites of small and large ruminants. Remarkably, *Teladorsagia circumcincta*, the most frequently found parasite in sheep in a recent study in Northern Germany [40], was not found on any of the OWC farms. Reports about *C. mentulatus* in domestic ruminants are rather rare [79–84], while there are several reports from wild ruminants [85–92], New World camelids [70, 93], and dromedaries [93, 94]. Interestingly, the only farm on which *C. mentulatus* predominated was the only farm that never treated its OWCs. Overall, there was a predominance of strongyle species that co-evolved with ruminants and not with OWCs, while many typical parasites of OWCs were absent. It is unclear what effects such untypical strongyle communities have on the health status of the camels. Unfortunately, quantitative data from parasite communities of OWCs in their original geographic range to be used for comparison are scarce, and we do not know how frequently species, such as *H. contortus*, occur in OWCs in (semi-)arid habitats. Moreover, there are currently no data available regarding species-specific clinical outcomes in OWCs depending on the composition of strongyle communities.

A small number of reads were not assigned to a specific species but only to the genus (unclassified *Haemonchus* and *Trichostrongylus*). This also occurred in previous nemabiome studies on ruminants and can be attributed to the small differences in the ITS-2 between species within the same strongyle genus [61]. New genetic variants not present in the sequence database or small PCR/sequencing errors might lead to the situation that an ASV cannot be assigned to a species anymore. However, the number of reads that remained unclassified was very low in all samples, and these numbers do not have any relevant effect on the major conclusions of the study.

The nemabiome approach is very powerful to characterize the strongyle communities of ruminants, for which the database has an excellent coverage of almost all relevant host species. It has recently also been applied to porcine samples, and the database also included all relevant species [30]. However, for more exotic species, such as wildlife or under-investigated livestock species, this might not be the case. Since OWCs in Germany shared all but one species (*C. mentulatus*) with species known from ruminant livestock, this was not a problem in the present study. However, the situation might be very different when samples from OWCs in their original endemic countries are analyzed. The method can in the future be used to compare, e.g., strongyle communities of dromedaries or Bactrian camels between different geographic and climatic zones, the effects of co-grazing ruminants on communities, parasite communities between age groups, and effects of stress caused by environmental factors or livestock-associated conditions, such as housing, feeding, and stocking density, as well as major utilization (milk, meat, sport, etc.).

The main limitation of the study is the fact that on the majority of the farms, the animals were not weighed, and the weight was estimated instead. In combination with the fact that none of the medicines was licensed for treatment of OWCs, this is why the study cannot prove resistance of the parasite communities to treatment. On two of the farms, late collection of post-treatment samples (day 21 on two farms) might also have some impact on the results, but the authors are confident that this effect was very minor. The ITS-2-based species identification also has some limitations. Within a genus, the number of polymorphisms that discriminate between species is very small [61, 95].

## Conclusions

The OWCs on the farms included in the present study showed poor treatment efficacy in response to benzimidazoles and macrocyclic lactones, while monepantel was fully active. The animals suffered from infections with *C. mentulatus* and several strongyle species common in domestic ruminants, particularly with *T. colubriformis* and *H. contortus.* Furthermore, the latter species showed low treatment efficacy against multiple drug classes, probably due to resistant parasite populations, although other factors such as the use of drugs not licensed for OWCs, and the estimation of body weight. Clear recommendations for dosages, availability of scales to determine animal weights before treatment exactly, and a regular quantitative determination of treatment efficacies are urgently needed to provide evidence-based husbandry and deworming strategy information for owners of OWCs in Europe. In addition, larger-scale investigations are required to obtain a clear picture of the extent of the problem of anthelmintic resistance in OWCs in Germany and other countries in Central Europe. Nemabiome data could, in the future, be used to determine the composition of strongyle communities in clinically conspicuous animals to determine which of the ruminant-adapted nematode species might cause severe disease in OWCs.

## Supplementary Information

Additional file 1.

Additional file 2.

Additional file 3.

## Data Availability

RNAseq data were deposited in the SRA section of Genbank^®^ under the BioProject number PRJNA1261439.

## Abbreviations

AAD: Aminoacetonitrile derivative
ABZ: Albendazole
BZ: Benzimidazole
CI: Confidence intervals
CrL: Credible limits
DOR: Doramectin
EPG: Eggs per gram
FBZ: Fenbendazole
FECR: Fecal egg count reduction
FECRT: Fecal egg count reduction test
GIN: Gastrointestinal nematodes
ITS-2: Internal transcribed spacer 2
IVM: Ivermectin
ML: Macrocyclic lactone
MON: Monepantel
MOX: Moxidectin
OWCs: Old World camels
