# Supplementary material for: Old world camels in Germany: parasitic nematode communities characterized by nemabiome analysis showed reduced anthelmintic efficacy according to the fecal egg count reduction test
Source: Parasit Vectors. 2025 Jul 24;18:294. doi: 10.1186/s13071-025-06930-9 (PMC12288319; doi:10.1186/s13071-025-06930-9)
Supplement: Supplementary file 1 — Additional file 1. [file 13071_2025_6930_MOESM1_ESM.pdf]

Old World camels in Germany: Parasitic nematode communities characterized nemabiome analysis show reduced anthelmintic efficacy according to the fecal egg count reduction test

Jenny Brachmann[1,2], Stefan Fiedler[3], Hannah Fischer[1,2,5], Jennifer S. Schmidt[1,2], Renate Radek[4], Georg von Samson-Himmelstjerna[1,2], Jürgen Krücken[1,2*]

[1]Institute for Parasitology and Tropical Veterinary Medicine, Freie Universität Berlin, Berlin, Germany

[2]Veterinary Centre for Resistance Research, Freie Universität Berlin, Berlin, German

[3]Federal Office of Consumer Protection and Food Safety, Berlin, German

[4]Evolutionary Biology, Institute of Biology, Freie Universität Berlin, Germany.

[5]Present address: German Federal Institute of Risk Assessment, Max-Dohrn-Str. 8-10, 10589 Berlin, Germany

Additional file 1

Questionaire for farms

Company name	
State	
Address<br>(Street, Zip Code, City)	
Vocational qualification	☐ farmer ☐ biologist ☐ veterinary ☐ zoo keeper ☐ other,____________
Experience with OWC (in years)	
Number of animals	☐ Dromedary ___________________________<br>☐ Bactrian camel _________________________<br>☐ Hybrid ________________________________
Other animal species kept on the farm	☐ lamas ☐ goats ☐ alpakas ☐ sheep ☐ horses ☐ cattle<br>☐ other, __________________________________
Other animal species that are at least temporarily/occasionally on the same areas	☐ lamas ☐ goats ☐ alpakas ☐ sheep ☐ horses ☐ cattle<br>☐ other, __________________________________
Number OWC on farm	
(1) bulls	
(2) bullocks	
(3) cows	
a) pregnant	
b) lactating	
(4) Young animals	
a) discontinued up to 3 years	
b) suckling	
Direction of use of the OWC?	☐ wool ☐ tourism ☐ breeding ☐ hobby keeping<br>☐ milk ☐ other, ___________________________
How many hours do your OWC spend every day in the barn?	winter ____________________________<br>summer ____________________________
How regularly is the barn mucked out?	☐ daily ☐ weekly ☐ monthly ☐ other, ________________________
How regularly is the barn disinfected?	☐ daily ☐ weekly ☐ monthly ☐ other, ________________________
How often is the litter completely changed?	☐ daily ☐ weekly ☐ monthly ☐ other, ________________________

Which material is used as litter in the barn?	☐ straw ☐ sawdust ☐ sand☒ Other, ________________________
How many hours a day do your animals spend on the pastures?	winter___________________________<br>summer___________________________
Is there a change of pastures, and if YES how regularly?	☐ yes, frequency: _______________________ ☐ no
Do other animal species also use the pastures (e.g. post-grazing)?	☐ yes, and _______________________ ☐ no
What are the OWC fed with?	☐ hay ☐ straw ☐ mowned gras ☐ silage ☐ boughs_trees, etc. from tree pruning ☐ pellets / concentrated feed
In which hight do you feed the OWC?	☐ floor level ☐ hay rack, elevated
Do your OWC receive additional minerals?	☐ yes ☐ no
Do your OWC receive additional salt?	☐ yes ☐ no
Do you deworm your OWC as an entire stock?	☐ yes ☐ no
How often do you deworm your OWC?	☐ never☐ only if necessary<br>☐ 1 to 2 times a year☐ 3 times a year
How often do you change the drug?	☐ with every treatment ☐ adjusted according to coproscopic results
How do you determine the body weight?	☐ weighted ☐ estimated
Are faecal examinations carried out regularly on your herd?	☐ yes, ☐1-2x a year ☐ more than 2x a year<br>☐ no

Questionaire: Individual animal

Company / camel owner	
Identification / name of OWC	
Camel species	☐ Dromedary ☐ Bactrian camel<br>☐ Tulu (hybrid)
Age	
Sex	☐ male ☐ female
Further details	☐ pregnant/lactating ☐ pregnant<br>☐ juvenile / suckling ☐ other, ________________________________
Last treatment	when: ____<br>drug:____
Origin of the individual	☐ born in the company ☐ purchased, from _____________________

Table S1 Overview of the sampled farms (B1 - B9) with dates of the first and second sampling, the date of deworming and arrival at the laboratory.

Farm	Pre-sampling	Arrival laboratory	Date of treatment	Post-treatment sampling	Arrival laboratory	Days between pre and post sample collection	Days between treatment and post treatment sample collection
B1	04.04.2023	05.04.2023	04.04.2023	18.04.2023	21.04.2023	14	14
B2	05.04.2023	05.04.2023	05.04.2023	18.04.2023	21.04.2023	13	13
B3	10.04.2023	11.04.2023	10.04.2023	24.04.2023	25.04.2023	14	14
B4	15.04.2023	16.04.2023	15.04.2023	01.05.2023	03.05.2023	16	16
B5	07.05.2023	07.05.2023	10.05.2023	29.05.2023	31.05.2023	22	19
B6	21.04.2023	21.04.2023					
B7	13.05.2023	14.05.2023	14.05.2023	04.06.2023	09.06.2023	22	21
B8	28.05.2023	29.05.2023	07.06.2023	21.06.2023	22.06.2023	24	14
B9	04.06.2023	04.06.2023	05.06.2023	20.06.2023	21.06.2023	16	15

Table S2 Primers used for Illumina library preparation.

Primer names	Primer sequences (5' -> 3')
Forward primers	
NC1_with_Illumina_Adapter_(0N)	TCGTCGGCAGCGTCAGATGTGTATAAGAGACAG**ACGTCTGGTTCAGGGTTGTT**
NC1_with_Illumina_Adapter_(1N)	TCGTCGGCAGCGTCAGATGTGTATAAGAGACAGN**ACGTCTGGTTCAGGGTTGTT**
NC1_with_Illumina_Adapter_(2N)	TCGTCGGCAGCGTCAGATGTGTATAAGAGACAGNN**ACGTCTGGTTCAGGGTTGTT**
NC1_with_Illumina_Adapter_(3N)	TCGTCGGCAGCGTCAGATGTGTATAAGAGACAGNNN**ACGTCTGGTTCAGGGTTGT**T
Reverse primers	
NC2_with_Illumina_Adapter_(0N)	GTCTCGTGGGCTCGGAGATGTGTATAAGAGACAG**TTAGTTTCTTTTCCTCCGCT**
NC2_with_Illumina_Adapter_(1N)	GTCTCGTGGGCTCGGAGATGTGTATAAGAGACAGN**TTAGTTTCTTTTCCTCCGC**T
NC2_with_Illumina_Adapter_(2N)	GTCTCGTGGGCTCGGAGATGTGTATAAGAGACAGNN**TTAGTTTCTTTTCCTCCGCT**
NC2_with_Illumina_Adapter_(3N)	GTCTCGTGGGCTCGGAGATGTGTATAAGAGACAGNNN**TTAGTTTCTTTTCCTCCGCT**

Illumina adapters are underlined. Sequences corresponding to the original NC1/NC2 primers are shown in bold.

Table S4 Total quantity of isolated strongyle nematode eggs per farm before and after anthelmintic treatment.

Farm	No. eggs pre-treatment	No. aliquots	No. eggs post-treatment	No. Aliquots
B1	9.000	2	8.500	3
B2	0[a]	3		
B3	39.500	3	3.000	2
B4	226.000	4	22.500	3
B5	71.500	4	37.500	3
B6	28.000	3		
B7	456.000	2	160.500	3
B8	104.000	2	41.000	2
B9	30.000	3		

[a]No eggs visible under the microscope but the PCR was positive.

Table S5 Read numbers per sample and statistics for data cleaning by filtering, denoising and merging.

Sample ID	Raw data	Cutadapt	Filtered	Denoised forward	Denoised reverse	Merged	No chimera	Percent lost
B1/pre	90127	88522	75012	74789	74941	74147	74019	17.9
B1/post	82243	80816	66525	66455	66427	65288	65177	20.8
B2/pre	86249	85026	71572	71522	71536	71077	59240	31.3
B3/pre	85700	84886	72195	71949	72130	71214	71214	16.9
B3/post	62616	61861	52829	52825	52823	52328	52328	16.4
B4/pre	105481	104500	88494	88430	88366	86426	86234	18.2
B4/post	76667	75796	63311	63253	63282	62721	62694	18.2
B5/pre	74265	73393	63029	63023	62884	61083	61004	17.9
B5/post	109683	108458	90589	90518	90559	89884	89881	18.1
B6/pre	85830	84941	72945	72940	72926	71818	71013	17.2
B7/pre	97315	96010	80810	80741	80765	79434	79032	18.8
B7/post	70275	69530	55429	55412	55355	54117	54098	23.0
B8/pre	81943	80901	67714	67652	67683	66862	66823	18.5
B8/post	89814	88801	74531	74494	74497	73747	73588	18.1
B9/pre	70922	69676	55885	55813	55836	55136	55097	22.3
