# Supplementary material for: Old world camels in Germany: parasitic nematode communities characterized by nemabiome analysis showed reduced anthelmintic efficacy according to the fecal egg count reduction test
Source: Parasit Vectors. 2025 Jul 24;18:294. doi: 10.1186/s13071-025-06930-9 (PMC12288319; doi:10.1186/s13071-025-06930-9)

Old World camels in Germany: Parasitic nematode communities characterized nemabiome analysis show reduced anthelmintic efficacy according to the fecal egg count reduction test

Jenny Brachmann[1,2], Stefan Fiedler[3], Hannah Fischer[1,2,5], Jennifer S. Schmidt[1,2], Renate Radek[4], Georg von Samson-Himmelstjerna[1,2], Jürgen Krücken[1,2*]

[1]Institute for Parasitology and Tropical Veterinary Medicine, Freie Universität Berlin, Berlin, Germany

[2]Veterinary Centre for Resistance Research, Freie Universität Berlin, Berlin, German

[3]Federal Office of Consumer Protection and Food Safety, Berlin, German

[4]Evolutionary Biology, Institute of Biology, Freie Universität Berlin, Germany.

[5]Present address: German Federal Institute of Risk Assessment, Max-Dohrn-Str. 8-10, 10589 Berlin, Germany

Additional file 3

```
(base) conda activate nemabiome
(nemabiome) R

R version 4.1.3 (2022-03-10) -- "One Push-Up"
Copyright (C) 2022 The R Foundation for Statistical Computing
Platform: x86_64-conda-linux-gnu (64-bit)

R ist freie Software und kommt OHNE JEGLICHE GARANTIE.
Sie sind eingeladen, es unter bestimmten Bedingungen weiter zu verbreiten.
Tippen Sie 'license()' or 'licence()' f_r Details dazu.

R ist ein Gemeinschaftsprojekt mit vielen Beitragenden.
Tippen Sie 'contributors()' f_r mehr Information und 'citation()',
um zu erfahren, wie R oder R packages in Publikationen zitiert werden k_nnen.

Tippen Sie 'demo()' f_r einige Demos, 'help()' f_r on-line Hilfe, oder
'help.start()' f_r eine HTML Browserschnittstelle zur Hilfe.
Tippen Sie 'q()', um R zu verlassen.

> > pkgs <- c("DECIPHER", "dada2", "ShortRead", "Biostrings", "ggplot2", "stringr",
"readr","phyloseq")
> lapply(pkgs, require, character.only = TRUE)
Fehler: Unerwartete(s) '>' in ">"
> pkgs <- c("DECIPHER", "dada2", "ShortRead", "Biostrings", "ggplot2", "stringr", "readr","phyloseq")
> lapply(pkgs, require, character.only = TRUE)
Lade n_tiges Paket: DECIPHER
Lade n_tiges Paket: Biostrings
Lade n_tiges Paket: BiocGenerics

Attache Paket: _BiocGenerics_

Die folgenden Objekte sind maskiert von _package:stats_:

    IQR, mad, sd, var, xtabs

Die folgenden Objekte sind maskiert von _package:base_:

    anyDuplicated, append, as.data.frame, basename, cbind, colnames,
    dirname, do.call, duplicated, eval, evalq, Filter, Find, get, grep,
    grepl, intersect, is.unsorted, lapply, Map, mapply, match, mget,
    order, paste, pmax, pmax.int, pmin, pmin.int, Position, rank,
    rbind, Reduce, rownames, sapply, setdiff, sort, table, tapply,
    union, unique, unsplit, which.max, which.min

Lade n_tiges Paket: S4Vectors
Lade n_tiges Paket: stats4

Attache Paket: _S4Vectors_
```

Die folgenden Objekte sind maskiert von _package:base_:

    expand.grid, I, unname

Lade n_tiges Paket: IRanges
Lade n_tiges Paket: XVector
Lade n_tiges Paket: GenomeInfoDb

Attache Paket: _Biostrings_

Das folgende Objekt ist maskiert _package:base_:

    strsplit

Lade n_tiges Paket: RSQLite
Lade n_tiges Paket: parallel
Lade n_tiges Paket: dada2
Lade n_tiges Paket: Rcpp
Lade n_tiges Paket: ShortRead
Lade n_tiges Paket: BiocParallel
Lade n_tiges Paket: Rsamtools
Lade n_tiges Paket: GenomicRanges
Lade n_tiges Paket: GenomicAlignments
Lade n_tiges Paket: SummarizedExperiment
Lade n_tiges Paket: MatrixGenerics
Lade n_tiges Paket: matrixStats

Attache Paket: _MatrixGenerics_

Die folgenden Objekte sind maskiert von _package:matrixStats_:

    colAlls, colAnyNAs, colAnys, colAvgsPerRowSet, colCollapse,
    colCounts, colCummaxs, colCummins, colCumprods, colCumsums,
    colDiffs, colIQRDiffs, colIQRs, colLogSumExps, colMadDiffs,
    colMads, colMaxs, colMeans2, colMedians, colMins, colOrderStats,
    colProds, colQuantiles, colRanges, colRanks, colSdDiffs, colSds,
    colSums2, colTabulates, colVarDiffs, colVars, colWeightedMads,
    colWeightedMeans, colWeightedMedians, colWeightedSds,
    colWeightedVars, rowAlls, rowAnyNAs, rowAnys, rowAvgsPerColSet,
    rowCollapse, rowCounts, rowCummaxs, rowCummins, rowCumprods,
    rowCumsums, rowDiffs, rowIQRDiffs, rowIQRs, rowLogSumExps,
    rowMadDiffs, rowMads, rowMaxs, rowMeans2, rowMedians, rowMins,
    rowOrderStats, rowProds, rowQuantiles, rowRanges, rowRanks,
    rowSdDiffs, rowSds, rowSums2, rowTabulates, rowVarDiffs, rowVars,
    rowWeightedMads, rowWeightedMeans, rowWeightedMedians,
    rowWeightedSds, rowWeightedVars

Lade n_tiges Paket: Biobase
Welcome to Bioconductor

```
    Vignettes contain introductory material; view with
    'browseVignettes()'. To cite Bioconductor, see
    'citation("Biobase")', and for packages 'citation("pkgname")'.


Attache Paket: _Biobase_

Das folgende Objekt ist maskiert _package:MatrixGenerics_:

    rowMedians

Die folgenden Objekte sind maskiert von _package:matrixStats_:

    anyMissing, rowMedians

Lade n_tiges Paket: ggplot2
Lade n_tiges Paket: stringr
Lade n_tiges Paket: readr
Lade n_tiges Paket: phyloseq

Attache Paket: _phyloseq_

Das folgende Objekt ist maskiert _package:SummarizedExperiment_:

    distance

Das folgende Objekt ist maskiert _package:Biobase_:

    sampleNames

Das folgende Objekt ist maskiert _package:GenomicRanges_:

    distance

Das folgende Objekt ist maskiert _package:IRanges_:

    distance

[[1]]
[1] TRUE

[[2]]
[1] TRUE

[[3]]
[1] TRUE

[[4]]
```

```
[1] TRUE

[[5]]
[1] TRUE

[[6]]
[1] TRUE

[[7]]
[1] TRUE

[[8]]
[1] TRUE

> lapply(pkgs, packageVersion)
[[1]]
[1] _2.22.0_

[[2]]
[1] _1.22.0_

[[3]]
[1] _1.52.0_

[[4]]
[1] _2.62.0_

[[5]]
[1] _3.3.5_

[[6]]
[1] _1.4.0_

[[7]]
[1] _2.1.2_

[[8]]
[1] _1.38.0_

> set.seed(202308)
> path = "/project_data/202305_Nemabiome_FU/sheep_goat/camel/rawdata_camel"
> fwd_files = sort(list.files(path, pattern = "R1", full.names = TRUE))
> rev_files = sort(list.files(path, pattern = "R2", full.names = TRUE))
> samples = str_extract(basename(fwd_files), "^[^_]+")
> samples
character(0)
> path = "/project_data/202305_Nemabiome_FU/data_Aug23/rawdata"
> fwd_files = sort(list.files(path, pattern = "R1", full.names = TRUE))
> rev_files = sort(list.files(path, pattern = "R2", full.names = TRUE))
```

```
> samples = str_extract(basename(fwd_files), "^[^_]+")
> samples
 [1] "23-0433-NK1pre-UNK"   "23-0434-NK2post-UNK"  "23-0435-SH1pre-UNK"
 [4] "23-0436-NH2post-UNK"  "23-0437-NH1pre-UNK"   "23-0438-OP1pre-UNK"
 [7] "23-0439-OP2post-UNK"  "23-0440-EFH1pre-UNK"  "23-0441-EFH2post-UNK"
[10] "23-0442-TPB1pre-UNK"  "23-0443-RS1pre-UNK"   "23-0444-RS2post-UNK"
[13] "23-0445-BK1pre-UNK"   "23-0446-BK2post-UNK"  "23-0447-BM1pre-UNK"
> names(fwd_files) <- samples
> names(rev_files) <- samples
> fwd_primer <- "ACGTCTGGTTCAGGGTTGTT"
> rev_primer <- "TTAGTTTCTTTTCCTCCGCT"
> fwd_primer_rev <- as.character(reverseComplement(DNAStringSet(fwd_primer)))
> rev_primer_rev <- as.character(reverseComplement(DNAStringSet(rev_primer)))
> count_primers <- function(primer, filename) {
  num_hits <- vcountPattern(primer, sread(readFastq(filename)), fixed = FALSE)
  return(sum(num_hits > 0))
}
> count_primers(fwd_primer, fwd_files[[1]])
[1] 87775
> count_primers(rev_primer, rev_files[[1]])
count_primers(fwd_primer, rev_files[[1]])
count_primers(rev_primer, fwd_files[[1]])
[1] 83620
[1] 553
[1] 559
> rawdata <- c()
> for (file in fwd_files)
{
data = readFastq(file)
length = as.integer(summary(data)['Length'])
rawdata = append(rawdata,length)
}
> track = data.frame(names(fwd_files), rawdata)
> cutadapt <- "/opt/NGS_Software/miniconda3/envs/nemabiome/bin/cutadapt"
> system2(cutadapt, args = "--version")
3.7
> cut_dir <- "/project_data/202305_Nemabiome/sheep_goat/camel/cutadapt"
> if (!dir.exists(cut_dir)) dir.create(cut_dir)
Warnmeldung:
In dir.create(cut_dir) :
  kann Verzeichnis '/project_data/202305_Nemabiome/sheep_goat/camel/cutadapt' nicht erzeugen.
Grund 'Datei oder Verzeichnis nicht gefunden'
> cut_dir <- "/project_data/202305_Nemabiome_FU/data_Aug23/cutadapt"
> if (!dir.exists(cut_dir)) dir.create(cut_dir)
> fwd_cut <- file.path(cut_dir, basename(fwd_files))
> rev_cut <- file.path(cut_dir, basename(rev_files))
> names(fwd_cut) <- samples
> names(rev_cut) <- samples
> cut_logs <- path.expand(file.path(cut_dir, paste0(samples, ".log")))
```

```
> cutadapt_args <- c("-g", fwd_primer, "-a", rev_primer_rev,
               "-G", rev_primer, "-A", fwd_primer_rev,
               "-n", 2, "--discard-untrimmed",
               "--minimum-length", 50)
> for (i in seq_along(fwd_files)) {
  system2(cutadapt,
        args = c(cutadapt_args,
               "-o", fwd_cut[i], "-p", rev_cut[i],
               fwd_files[i], rev_files[i]),
        stdout = cut_logs[i])
}
Done          00:00:08       90,127 reads @  91.7 _s/read;   0.65 M reads/minute
Done          00:00:07       82,243 reads @  92.4 _s/read;   0.65 M reads/minute
Done          00:00:07       86,249 reads @  92.3 _s/read;   0.65 M reads/minute
Done          00:00:07       85,700 reads @  90.0 _s/read;   0.67 M reads/minute
Done          00:00:05       62,616 reads @  90.6 _s/read;   0.66 M reads/minute
Done          00:00:09      105,481 reads @  92.1 _s/read;   0.65 M reads/minute
Done          00:00:07       76,667 reads @  93.8 _s/read;   0.64 M reads/minute
Done          00:00:06       74,265 reads @  90.5 _s/read;   0.66 M reads/minute
Done          00:00:09      109,683 reads @  89.0 _s/read;   0.67 M reads/minute
Done          00:00:07       85,830 reads @  90.0 _s/read;   0.67 M reads/minute
Done          00:00:08       97,315 reads @  91.3 _s/read;   0.66 M reads/minute
Done          00:00:06       70,275 reads @  94.7 _s/read;   0.63 M reads/minute
Done          00:00:07       81,943 reads @  94.0 _s/read;   0.64 M reads/minute
Done          00:00:08       89,814 reads @  92.7 _s/read;   0.65 M reads/minute
Done          00:00:06       70,922 reads @  92.2 _s/read;   0.65 M reads/minute
> cutted <- c()
> for (file in fwd_cut)
{
data = readFastq(file)
length = as.integer(summary(data)['Length'])
cutted = append(cutted,length)
}
> track["cutadapt"] = cutted
> plotQualityProfile(fwd_cut) + ggtitle("Forward")
Warnmeldung:
`guides(<scale> = FALSE)` is deprecated. Please use `guides(<scale> = "none")` instead.
> plotQualityProfile(rev_cut) + ggtitle("Reverse")
Warnmeldung:
`guides(<scale> = FALSE)` is deprecated. Please use `guides(<scale> = "none")` instead.
> filt_dir <- "/project_data/202305_Nemabiome_FU/data_Aug23/filtered"
> if (!dir.exists(filt_dir)) dir.create(filt_dir)
> fwd_filt <- file.path(filt_dir, basename(fwd_files))
> rev_filt <- file.path(filt_dir, basename(rev_files))
> names(fwd_filt) <- samples
> names(rev_filt) <- samples
> filtered_out <- filterAndTrim(
  fwd = fwd_cut,
  filt = fwd_filt,
```

```
  rev = rev_cut,
  filt.rev = rev_filt,
  maxEE = c(2, 5),
  truncQ = 2,
  rm.phix = TRUE,
  compress = TRUE,
  multithread = TRUE
)
> plotQualityProfile(fwd_filt[1:2]) + ggtitle("Forward filtered")
Warnmeldung:
`guides(<scale> = FALSE)` is deprecated. Please use `guides(<scale> = "none")` instead.
> plotQualityProfile(rev_filt[1:2]) + ggtitle("Reverse filtered")
Warnmeldung:
`guides(<scale> = FALSE)` is deprecated. Please use `guides(<scale> = "none")` instead.
> dev.off()
null device
        1
> track["filtered"] <- filtered_out[,2]
> as.matrix(filtered_out[,2]/filtered_out[,1]*100)
                                             [,1]
23-0433-NK1pre-UNK_S42_L001_R1_001.fastq.gz   84.73826
23-0434-NK2post-UNK_S43_L001_R1_001.fastq.gz  82.31662
23-0435-SH1pre-UNK_S44_L001_R1_001.fastq.gz   84.17660
23-0436-NH2post-UNK_S45_L001_R1_001.fastq.gz  85.04936
23-0437-NH1pre-UNK_S46_L001_R1_001.fastq.gz   85.39952
23-0438-OP1pre-UNK_S47_L001_R1_001.fastq.gz   84.68325
23-0439-OP2post-UNK_S48_L001_R1_001.fastq.gz  83.52815
23-0440-EFH1pre-UNK_S49_L001_R1_001.fastq.gz  85.87876
23-0441-EFH2post-UNK_S50_L001_R1_001.fastq.gz 83.52450
23-0442-TPB1pre-UNK_S51_L001_R1_001.fastq.gz  85.87726
23-0443-RS1pre-UNK_S52_L001_R1_001.fastq.gz   84.16832
23-0444-RS2post-UNK_S53_L001_R1_001.fastq.gz  79.71955
23-0445-BK1pre-UNK_S54_L001_R1_001.fastq.gz   83.69983
23-0446-BK2post-UNK_S55_L001_R1_001.fastq.gz  83.93036
23-0447-BM1pre-UNK_S56_L001_R1_001.fastq.gz   80.20696
> err_fwd <- learnErrors(fwd_filt, multithread = TRUE)
118958570 total bases in 426627 reads from 6 samples will be used for learning the error rates.
> err_rev <- learnErrors(rev_filt, multithread = TRUE)
119049813 total bases in 426627 reads from 6 samples will be used for learning the error rates.
> plotErrors(err_fwd, nominalQ = TRUE)
Warnmeldung:
Transformation introduced infinite values in continuous y-axis
> plotErrors(err_rev, nominalQ = TRUE)
Warnmeldung:
Transformation introduced infinite values in continuous y-axis
> dev.off()
null device
        1
> dada_fwd <- dada(fwd_filt, err = err_fwd, multithread = TRUE)
```

```
Sample 1 - 75012 reads in 8054 unique sequences.
Sample 2 - 66525 reads in 8474 unique sequences.
Sample 3 - 71572 reads in 8548 unique sequences.
Sample 4 - 72195 reads in 6316 unique sequences.
Sample 5 - 52829 reads in 5404 unique sequences.
Sample 6 - 88494 reads in 8922 unique sequences.
Sample 7 - 63311 reads in 6155 unique sequences.
Sample 8 - 63029 reads in 6623 unique sequences.
Sample 9 - 90589 reads in 6700 unique sequences.
Sample 10 - 72945 reads in 9119 unique sequences.
Sample 11 - 80810 reads in 8466 unique sequences.
Sample 12 - 55429 reads in 7446 unique sequences.
Sample 13 - 67714 reads in 7646 unique sequences.
Sample 14 - 74531 reads in 8493 unique sequences.
Sample 15 - 55885 reads in 5563 unique sequences.
> dada_rev <- dada(rev_filt, err = err_rev, multithread = TRUE)
Sample 1 - 75012 reads in 36078 unique sequences.
Sample 2 - 66525 reads in 35384 unique sequences.
Sample 3 - 71572 reads in 36067 unique sequences.
Sample 4 - 72195 reads in 32054 unique sequences.
Sample 5 - 52829 reads in 24381 unique sequences.
Sample 6 - 88494 reads in 41865 unique sequences.
Sample 7 - 63311 reads in 30565 unique sequences.
Sample 8 - 63029 reads in 29431 unique sequences.
Sample 9 - 90589 reads in 41632 unique sequences.
Sample 10 - 72945 reads in 34290 unique sequences.
Sample 11 - 80810 reads in 38608 unique sequences.
Sample 12 - 55429 reads in 31762 unique sequences.
Sample 13 - 67714 reads in 33130 unique sequences.
Sample 14 - 74531 reads in 37151 unique sequences.
Sample 15 - 55885 reads in 30279 unique sequences.
> getN <- function(x) sum(getUniques(x))
> track["denoised_fwd"] <- sapply(dada_fwd, getN)
track["denoised_rev"] <- sapply(dada_rev, getN)
> mergers <- mergePairs(
  dadaF = dada_fwd,
  dadaR = dada_rev,
  derepF = fwd_filt,
  derepR = rev_filt,
  maxMismatch = 1,
  verbose=TRUE
)
74147 paired-reads (in 33 unique pairings) successfully merged out of 74751 (in 74 pairings) input.
Duplicate sequences in merged output.
65288 paired-reads (in 37 unique pairings) successfully merged out of 66376 (in 102 pairings) input.
Duplicate sequences in merged output.
71077 paired-reads (in 45 unique pairings) successfully merged out of 71500 (in 83 pairings) input.
Duplicate sequences in merged output.
71214 paired-reads (in 12 unique pairings) successfully merged out of 71890 (in 29 pairings) input.
```

```
Duplicate sequences in merged output.
52328 paired-reads (in 12 unique pairings) successfully merged out of 52821 (in 29 pairings) input.
Duplicate sequences in merged output.
86426 paired-reads (in 39 unique pairings) successfully merged out of 88326 (in 122 pairings) input.
Duplicate sequences in merged output.
62721 paired-reads (in 21 unique pairings) successfully merged out of 63232 (in 56 pairings) input.
Duplicate sequences in merged output.
61083 paired-reads (in 21 unique pairings) successfully merged out of 62881 (in 76 pairings) input.
Duplicate sequences in merged output.
89884 paired-reads (in 22 unique pairings) successfully merged out of 90500 (in 56 pairings) input.
Duplicate sequences in merged output.
71818 paired-reads (in 38 unique pairings) successfully merged out of 72924 (in 97 pairings) input.
Duplicate sequences in merged output.
79434 paired-reads (in 37 unique pairings) successfully merged out of 80709 (in 126 pairings) input.
Duplicate sequences in merged output.
54117 paired-reads (in 28 unique pairings) successfully merged out of 55354 (in 108 pairings) input.
Duplicate sequences in merged output.
66862 paired-reads (in 22 unique pairings) successfully merged out of 67629 (in 75 pairings) input.
Duplicate sequences in merged output.
73747 paired-reads (in 23 unique pairings) successfully merged out of 74471 (in 77 pairings) input.
Duplicate sequences in merged output.
55136 paired-reads (in 32 unique pairings) successfully merged out of 55792 (in 79 pairings) input.
Duplicate sequences in merged output.
> seqtab <- makeSequenceTable(mergers)
Duplicate sequences detected and merged.
Duplicate sequences detected and merged.
Duplicate sequences detected and merged.
Duplicate sequences detected and merged.
Duplicate sequences detected and merged.
Duplicate sequences detected and merged.
Duplicate sequences detected and merged.
Duplicate sequences detected and merged.
Duplicate sequences detected and merged.
Duplicate sequences detected and merged.
Duplicate sequences detected and merged.
Duplicate sequences detected and merged.
Duplicate sequences detected and merged.
Duplicate sequences detected and merged.
Duplicate sequences detected and merged.
> dim(seqtab)
[1] 15 69
> track["merged"] <- sapply(mergers, getN)
Duplicate sequences detected and merged.
Duplicate sequences detected and merged.
Duplicate sequences detected and merged.
Duplicate sequences detected and merged.
Duplicate sequences detected and merged.
Duplicate sequences detected and merged.
Duplicate sequences detected and merged.
```

```
Duplicate sequences detected and merged.
Duplicate sequences detected and merged.
Duplicate sequences detected and merged.
Duplicate sequences detected and merged.
Duplicate sequences detected and merged.
Duplicate sequences detected and merged.
Duplicate sequences detected and merged.
Duplicate sequences detected and merged.
> seqtab_nochim <- removeBimeraDenovo(seqtab, method = "consensus", multithread = TRUE,
verbose = TRUE)
Identified 22 bimeras out of 69 input sequences.
> dim(seqtab_nochim)
[1] 15 47
> track["no chimera"] <- rowSums(seqtab_nochim)
> table(nchar(getSequences(seqtab_nochim)))

189 281 283 287 288 291 292
  1  18   6   1  12   8   1
> lost_reads <- 100 - track["no chimera"]/track["rawdata"]*100
> names(lost_reads) <- NULL
> track["perc lost"] <- lost_reads
> colnames(track["perc lost"]) <- "perc lost"
> summary(track["perc lost"])
   perc lost
 Min.   :16.43
 1st Qu.:17.86
 Median :18.23
 Mean   :19.57
 3rd Qu.:19.77
 Max.   :31.32
> summary(track["no chimera"])
   no chimera
 Min.   :52328
 1st Qu.:60122
 Median :66823
 Mean   :68096
 3rd Qu.:73804
 Max.   :89881
> train <-
readDNAStringSet("/data/sfiedler/databases_SF/DB_Nematode_ITS2_v1_5_0_SF/idtaxa.fasta")
Fehler in .Call2("new_input_filexp", filepath, PACKAGE = "XVector") :
  cannot open file '/data/sfiedler/databases_SF/DB_Nematode_ITS2_v1_5_0_SF/idtaxa.fasta'
> train <-
readDNAStringSet("/data/sfiedler/databases_SF/DB_Nematode_ITS2_v1_5_0_SF/idtaxa_SF.fasta")
> tax <- read_tsv("/data/sfiedler/databases_SF/DB_Nematode_ITS2_v1_5_0_SF/idtaxa_SF.tax")
Rows: 3976 Columns: 5
── Column specification
────────────────────────────────────────────────────────
```

```
Delimiter: "\t"
chr (2): Name, Rank
dbl (3): Index, Parent, Level

_ Use `spec()` to retrieve the full column specification for this data.
_ Specify the column types or set `show_col_types = FALSE` to quiet this message.
> trainingSet <- LearnTaxa(train, names(train), tax)
  |=======================================================================| 100%

Time difference of 233.92 secs

> dna <- DNAStringSet(getSequences(seqtab_nochim))
> idtaxa <- IdTaxa(dna,
           trainingSet,
           strand = "both",
           threshold = 60,
           bootstraps = 100,
           processors = NULL,
           verbose = TRUE,
           type = "extended")
  |=======================================================================| 100%

Time difference of 3.03 secs

> samp_data <-  data.frame(
  row.names = samples,
  sample = samples
)
> asvs <- paste0("ASV_", 1:length(dna))
> taxid <- t(sapply(idtaxa, function(x) setNames(x$taxon, x$rank)))[, -1]
> ranks <- c("rootrank", "superkingdom", "kingdom", "phylum", "class", "order", "family", "genus",
"species")
> taxid2 <- t(sapply(idtaxa, function(x) {m <- match(ranks, x$rank); taxa <- x$taxon[m]; taxa}))
> taxid3 <- as.matrix(sapply(idtaxa, function(x) { m<-match(ranks, x$rank); taxa <- x$taxon[m];
paste(taxa[-1], collapse="; ")}))
> colnames(taxid3) <- "Taxonomic classification"
> rownames(taxid3) <- asvs
> colnames(seqtab_nochim) <- asvs
> names(dna) <- asvs
> ps = phyloseq(
  otu_table(seqtab_nochim, taxa_are_rows = FALSE),
  tax_table(taxid3),
  sample_data(samp_data),
  dna
)
> ps
phyloseq-class experiment-level object
```

```
otu_table()   OTU Table:         [ 47 taxa and 15 samples ]
sample_data() Sample Data:       [ 15 samples by 1 sample variables ]
tax_table()   Taxonomy Table:    [ 47 taxa by 1 taxonomic ranks ]
refseq()      DNAStringSet:      [ 47 reference sequences ]
> filter <- phyloseq::genefilter_sample(ps, filterfun_sample(function(x) x >= 10))
> ps_filt <- prune_taxa(filter, ps)
> ps_filt
phyloseq-class experiment-level object
otu_table()   OTU Table:         [ 45 taxa and 15 samples ]
sample_data() Sample Data:       [ 15 samples by 1 sample variables ]
tax_table()   Taxonomy Table:    [ 45 taxa by 1 taxonomic ranks ]
refseq()      DNAStringSet:      [ 45 reference sequences ]
> asv_table <- cbind(t(otu_table(ps)),taxid3)
> write.table(asv_table, file="asv_table_w_taxonomy.tsv", sep="\t", quote=FALSE)
> writeFasta(dna, file="asv_sequences.fasta")
> write.table(track, file="filtering_stats.txt", sep="\t", quote=FALSE)
> q()
Save workspace image? [y/n/c]: y
```

Quality control plot R1

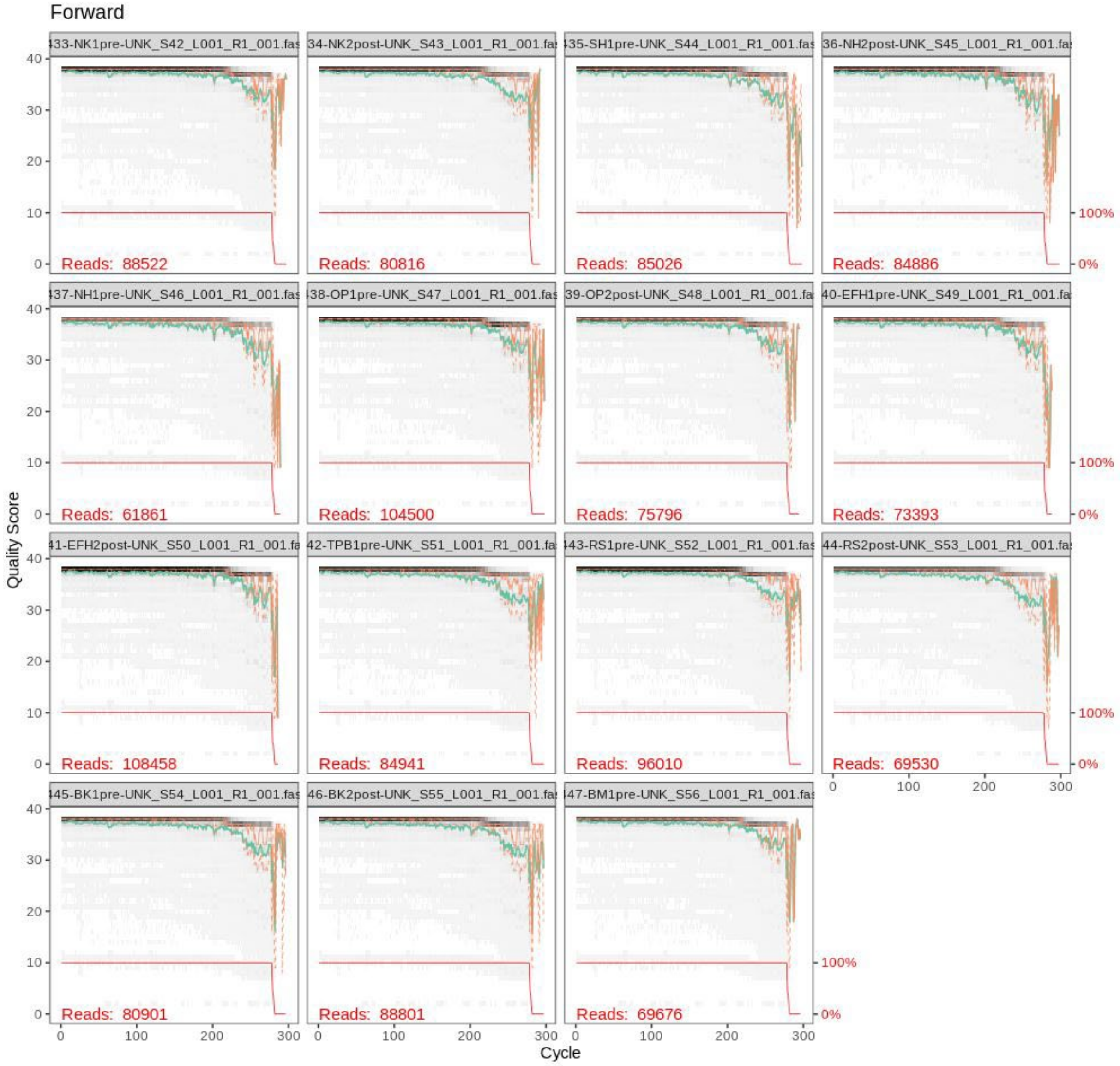

Quality control plot R2

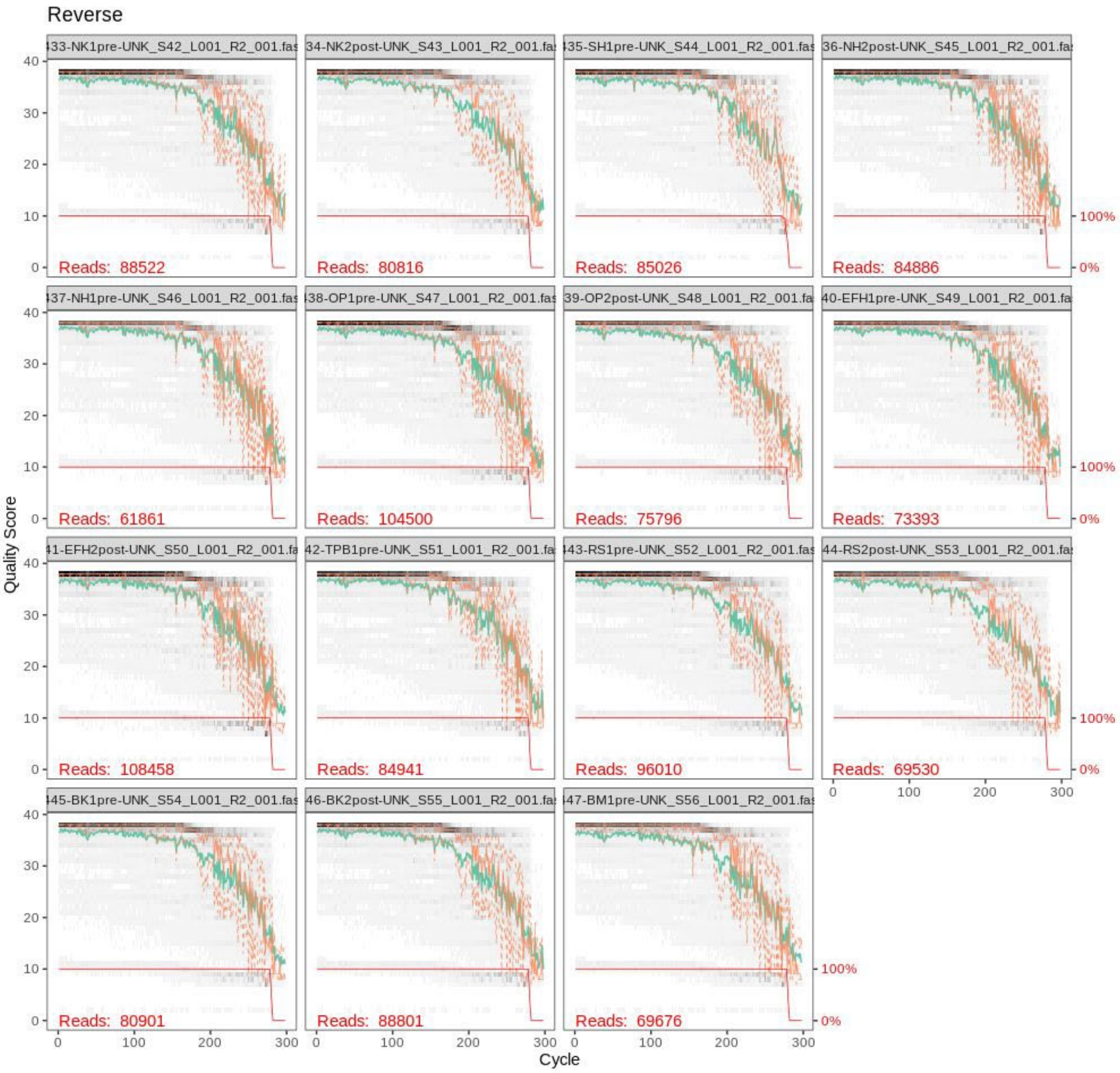

Quality control plot R1 after filtering

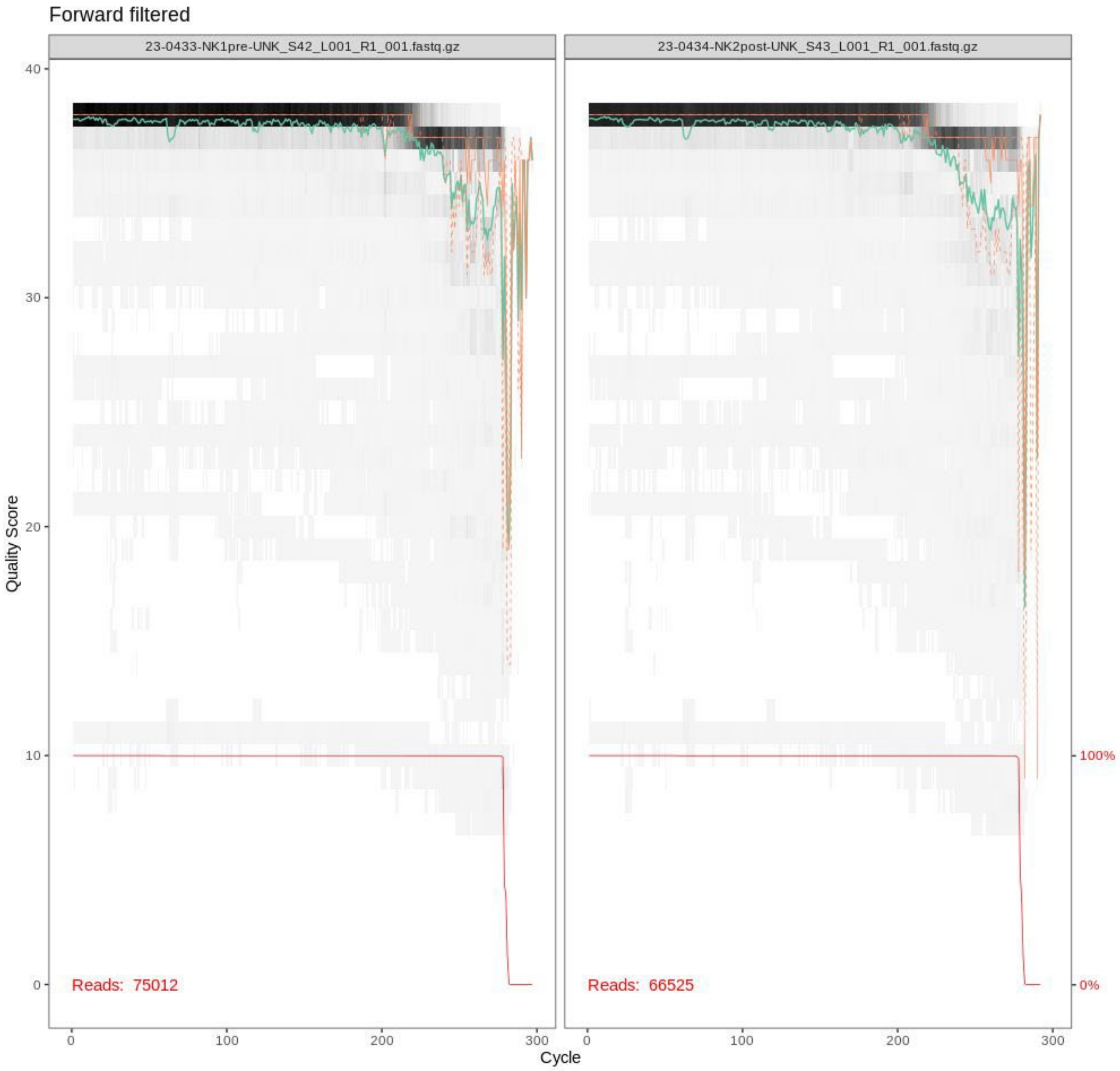

Quality control plot R2 after filtering

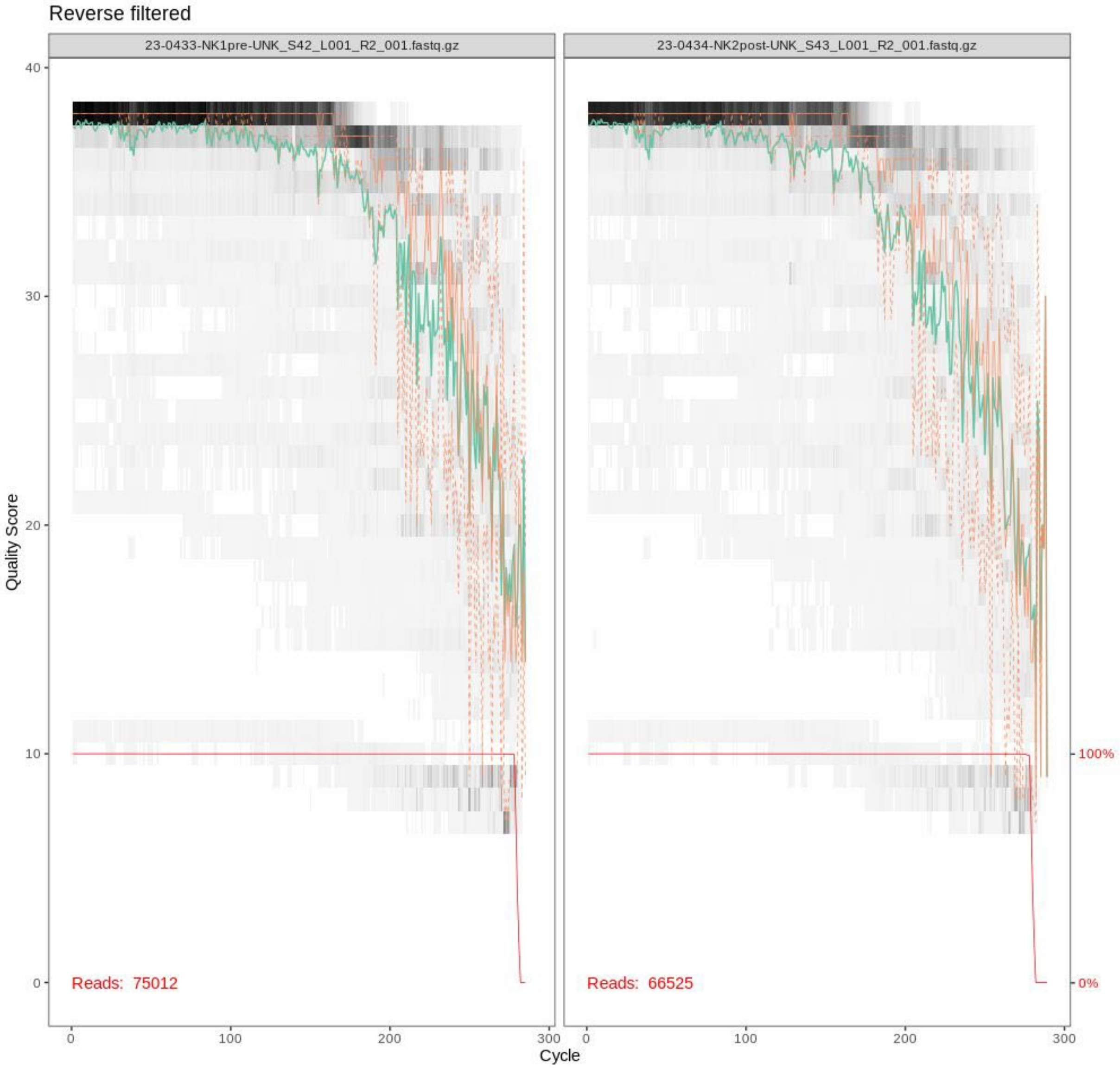

Error plots R1

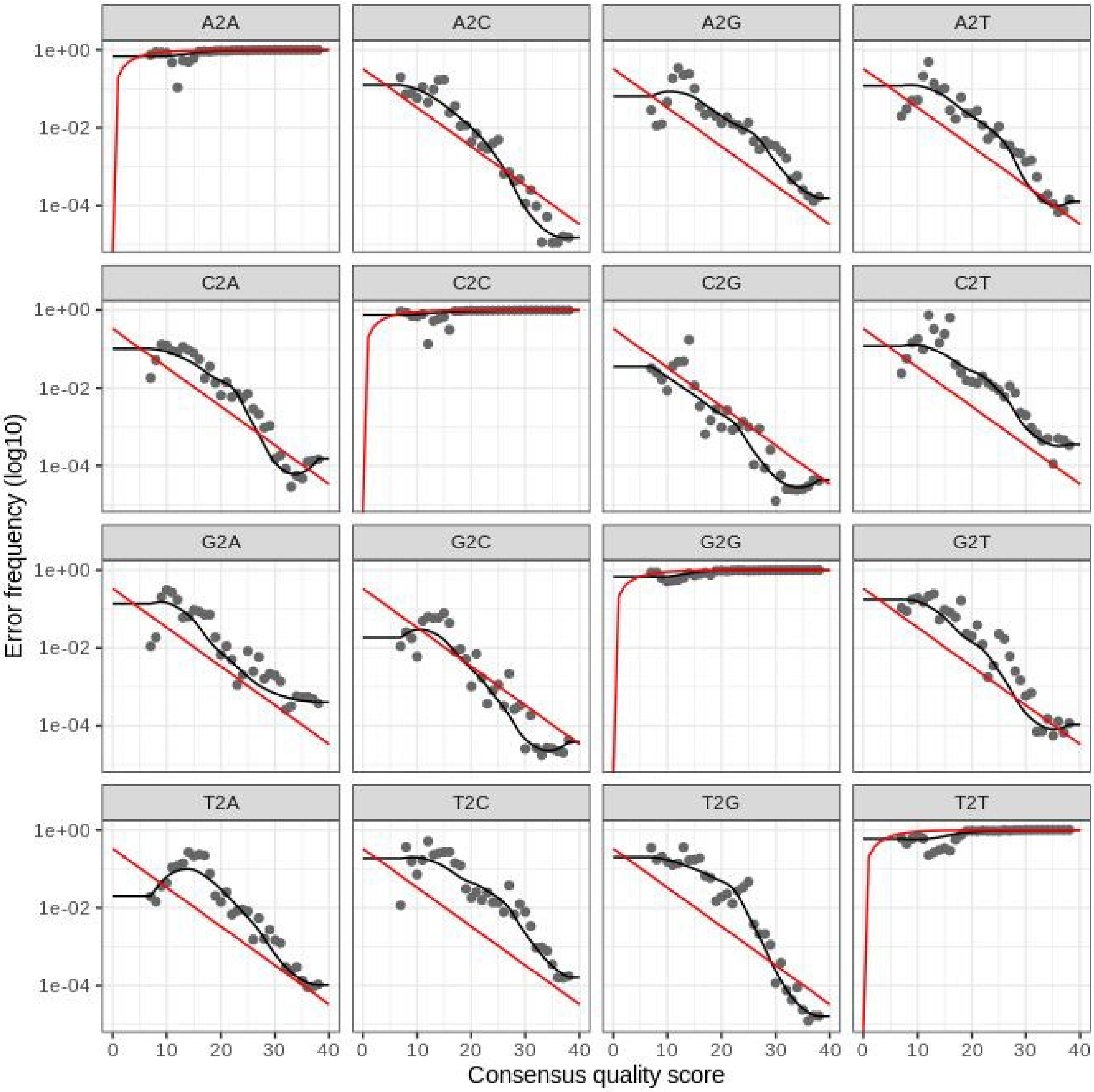

Error plots R2

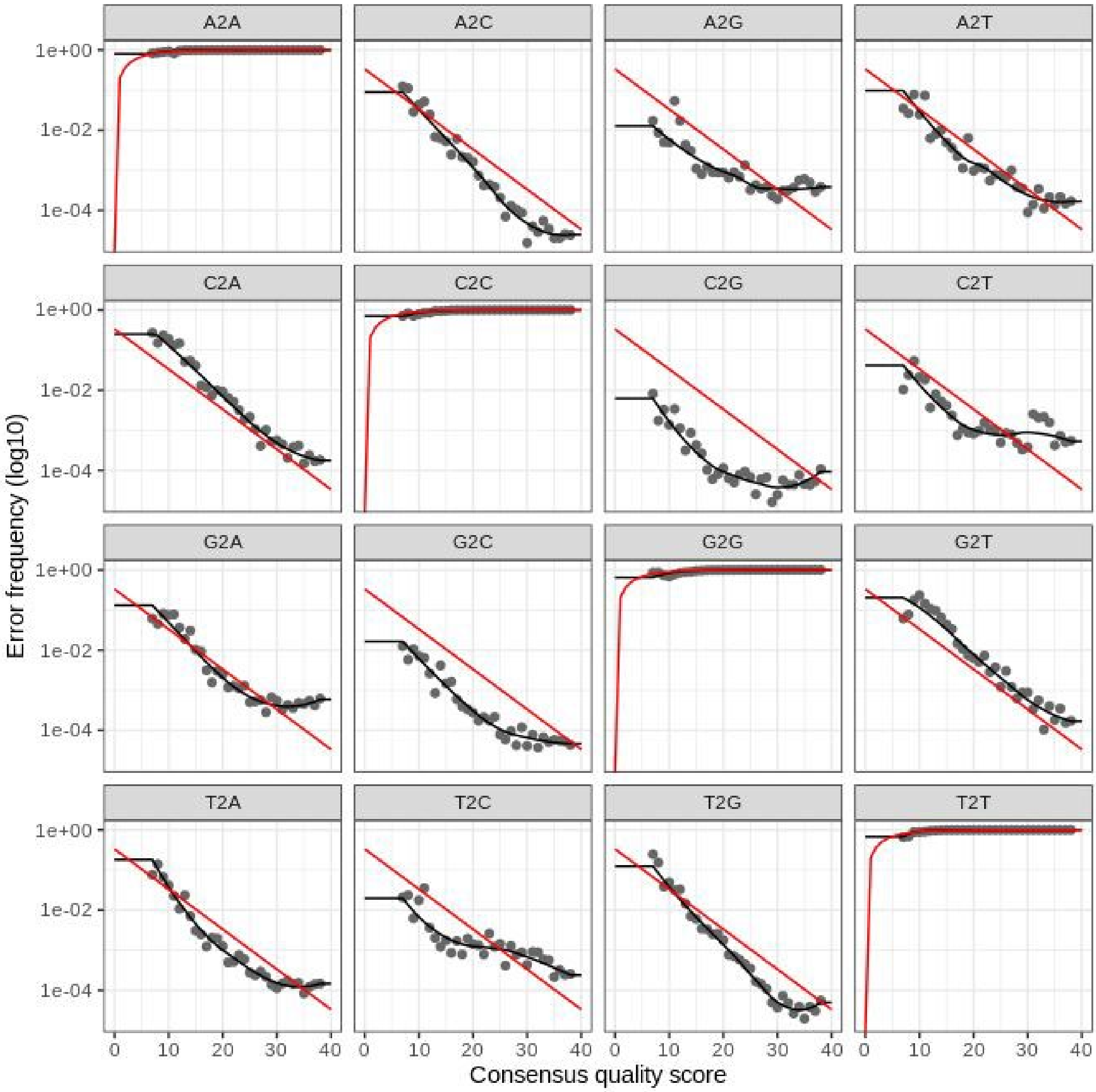

Supplement: Supplementary file 3 — Additional file 3. [file 13071_2025_6930_MOESM3_ESM.pdf]
